# Behaviour Recognition with Kinodynamic Planning Over Continuous Domains

**DOI:** 10.3389/frai.2021.717003

**Published:** 2021-10-25

**Authors:** Grady Fitzpatrick, Nir Lipovetzky, Michael Papasimeon, Miquel Ramirez, Mor Vered

**Affiliations:** ^1^ The University of Melbourne, Parkville, VIC, Australia; ^2^ Defence Science and Technology Group (DSTG), Edinburgh, SA, Australia; ^3^ Monash University, Melbourne, VIC, Australia

**Keywords:** behaviour recognition, aerial maneuvering, online recognition, plan recognition as planning, model predictive control (MPC)

## Abstract

We investigate the application of state-of-the-art goal recognition techniques for *behaviour* recognition over complex continuous domains using model predictive control (MPC) for trajectory generation. We formally define the problem of kinodynamic behaviour recognition and establish a set of baseline behaviours and performance measures in the complex domain of unmanned aerial maneuvers. We evaluate how well our approach performs over a range of standard aerial maneuvers and representative initial configurations of varying complexity. The work also highlights future research directions in compound model-based behaviour recognition and team behaviour recognition where multiple agents may be acting simultaneously.

## 1 Introduction

Behavior recognition is the problem of recognizing the behaviour of an agent from an incomplete trace of observations. This problem is closely related to plan and/or goal recognition (Sukthankar et al., 2014). While *plan* recognition involves highly complex reasoning to infer which plan best explains a sequence of observations, and *goal* recognition reasons about which goal best explains a sequence of observations, the problem of behaviour recognition is far more general. In behaviour recognition we reason about which *behaviour* explains a sequence of observations, behaviours being more loosely connected with specific domain instances. The same behaviour could be executed in different initial conditions and different instances of the environment, generating a range of different sequences of observations. Behaviours can be understood as generalized plans, which can be expressed as policies, algorithmic programs, or logical specifications (Srivastava et al., 2011; Jiménez et al., 2019).

Behaviour recognition is a widely researched problem with a wide array of implementations relevant for both adversarial opponent behaviour recognition (Kabanza et al., 2010) and behaviour recognition within a team of coordinated agents (Oh et al., 2014). Within the context of a team, behaviour recognition is core to realizing multi-agent coordinated behaviour. This is owing to the fact that in many instances inter-agent interaction *via* direct communication could be limited due to faulty devices, network capacity, time limitation, or security concerns (Heinze et al., 1999; Ilachinski, 2004; Oh et al., 2014). The identification and characterization of behaviours in complex, real-time continuous environments is a challenging problem which has to account for an unknown sequence of incoming observations and an infinite number of state possibilities.

Leading approaches to behaviour recognition leverage different means to discretize the states of continuous environments. These include using linear temporal logic (LTL) formulas (Calvanese et al., 2002; Pereira et al., 2021), using behaviour trees (Colledanchise et al., 2019; Sitanskiy et al., 2020) and Fuzzy Finite State Machine (FFSM) models (Mohmed et al., 2020) among others. However, these means of discretization imply that there is no notion of progress *between* two consecutive states. An agent can either be in one state or in the consecutive one, but not in between.

To address this challenge we develop and implement a novel behaviour recognition model which is able to differentiate between a range of complex multi-agent behaviours over *continuous* domains using model predictive control (MPC) for trajectory generation. We begin by providing a new, formal definition for the kinodynamic behaviour recognition problem in continuous domains whereby behaviours are characterized through stage and terminal costs. In this manner our model is uniquely able to cast the problem of recognising behaviours to that of seeking which *cost functions*, composing stage and terminal cost terms, are the best explanations for a given sequence of observations. We follow by empirically evaluating our approach on the real-time, continuous environment of aerial combat whereby we define and are able to differentiate between a range of five challenging aerial maneuvers.

Aerial combat is a highly complex, continuous domain: for each agent, its position, orientation, linear velocity and acceleration (thrust) need to be be tracked and trajectories span large periods of time covering long, continuous distances. Planning and recognition in these real world domains often calls for rapid responses to changing conditions and frequently requires planning in the absence of reliable data (Borck et al., 2015a).

To perform real-time behaviour recognition, we solve the problem as online *behavior* recognition in continuous domains. We tested the proposed approach over a benchmark consisting of many partial trajectories implementing a range of five complex aerial maneuvers, inherently hard to distinguish and unique to the challenging unmanned aerial maneuvers domain; flying straight, pure pursuit, lead pursuit, stern conversion and offensive flight. We were able to achieve a True Positive rate of 100% recognition and a False Negative rate of 2% while also evaluating the Single True Positive rates whereby the correct behaviour was recognized alone. Our results enable us to provide more insight as to the similarities of the behaviours and the challenges of the aerial maneuver domain.

The rest of the paper is constructed as follows; Section 2 discusses existing behaviour recognition research with particular focus on the aerial maneuvering domain. Section 3 provides a formal definition of the problem of behaviour recognition with online kinodynamic planning. Section 4 discusses the domain of aerial maneuvering and its modelling within the context of the ACE0 multi-agent-based-simulation environment. Section 5 describes the experimental setup used in this study and Section 6 presents and analyses the results and concludes the paper by briefly describing potential future work directions.

## 2 Related Work

The problem of behaviour recognition is closely linked to that of goal and/or plan recognition; recognizing a goal or intention without complete knowledge about any actions taken to achieve or further the former (Schmidt et al., 1978; Sukthankar et al., 2014; Van-Horenbeke and Peer, 2021). One approach towards solving the problem of goal recognition relies on the use of a dedicated *plan library* as the basis for the recognition process (Heinze et al., 1999). The plan library efficiently represents all known plans to achieve known goals. In this manner observations are matched against existing plans within the library to determine the most likely plan candidate. This approach is also prevalent among behaviour recognition research. Heinze (2004) investigated a range of software design patterns for implementing intention recognition in multi-agent systems including plan-library based approaches; Menager et al. (2017) focused on goal recognition of a team member’s goals and Azarewicz et al. (1986) focuses on plan recognition in the tactical air domain using a blackboard based cognitive architecture.

When it comes to behaviour recognition, the recognition of aircraft behaviour *via* the analysis of an aircraft’s trajectory is important in both civil and military aviation. In civil aviation, determining an aircraft’s behaviour is especially important in times where communication with the aircrew has been disrupted due to an emergency that is either accidental or explicitly malicious. In this case, an aircraft’s trajectory relative to known landmarks such as airports, way-points and known flight paths can be used to see if an aircraft is behaving in an anomalous manner. In military aviation, where uncertainty and the fog of war are ever present, behaviour recognition *via* radar surveillance (or other sensors) is important, not only for the purposes of early warning but also for enhancing situational awareness to determine the next course of action. This is of particular concern in the domain of air combat where an opponent is by definition non-cooperative and adversarial. In order to select a viable course of action, predicting the behaviour of an opponent aircraft (or team of aircraft) becomes critical to the success of a mission.

In the air combat domain, a behaviour could refer to an aircraft trajectory, a stand-alone maneuver, a maneuver relative to another aircraft or an individual or team tactic. Second, the type of agent reasoning model being used in a constructive simulation has the potential to influence if we are recognising an opponent’s behaviour, goals or intentions. For example, a number of air combat constructive simulations have been built using the beliefs-desires-intention (BDI) model (Rao and Georgeff, 1991) of agent reasoning. Since the BDI model explicitly models mental states such as beliefs, desires, plans and intentions one might consider the recognition of the mental states of other agents (in particular those of opponents or adversaries). (Rao et al., 1994) specifically discuss BDI mental state recognition in the air combat domain. These BDI models are often used in constructive multi agent simulations, with no human participants.

In contrast, human-in-the-loop flight simulators are often used to train both civil and military pilots augmenting and complementing real flight hours. A constructive simulation environment however, is typically used in the field of military operations research to study questions pertaining to aircraft procurement and acquisition, tactics and concept of operations development. Constructive simulation environments are often implemented as multi-agent-based simulations (Heinze et al., 2002) where agents represent the decision making processes of pilots or other aircrew (Heinze et al., 2008). In this work, unlike most state-of-the-art simulators, the behaviour recognition process has been explicitly modelled. This allows a single pilot agent (or a team of agents) to recognise what the opponent is doing and to ultimately select the appropriate course of action.

Within the context of complex, continuous domains, such as aerial combat, the earlier plan-library-based approaches fall short, as there are an infinite number of ways to achieve each behaviour, resulting in a potentially infinite number of plan representations. Each approach currently used for behaviour recognition deals differently with the problem of continuous domains. One approach characterizes behaviours through the use of linear temporal logic (LTL) formulas which aim to encode paths over time by using a finite set of propositional variables and logical and temporal mode operators (Calvanese et al., 2002; Pereira et al., 2021). Another approach uses behaviour trees as a modular representation describing switching between a finite set of tasks in a representation widely adopted to express expert knowledge (Colledanchise et al., 2019; Sitanskiy et al., 2020). A more recent approach uses a combination of Fuzzy Finite State Machine (FFSM) models and neural networks to represent behaviours, aiming to recognize the parameters used for generating the fuzzy logic that governs the transitions between the system’s states (Mohmed et al., 2020).

**FIGURE 1:**
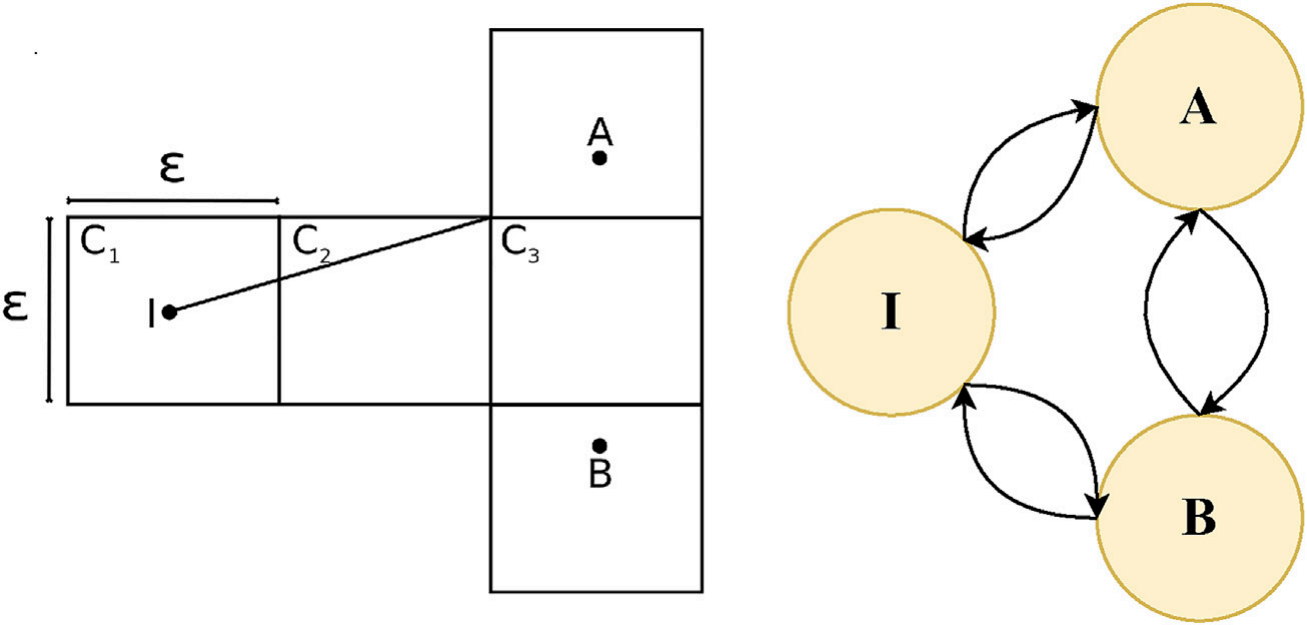
**(Left)** Discretized goal recognition problem replicated with permission from Kaminka et al. (2018). **(Right)** Same goal recognition problem extended as a Finite State Machine (FSM).

In order to define the transition between different states of a problem with continuous dynamics, all of these approaches adopt some means, either implicit or explicit, of discretization of states. This discretization implies that there is no notion of progress *in between* two consecutive states. An agent can either be in one state or in the consecutive state, but not in between. Figure 1, left, shows an intuitive, 2D, continuous, goal recognition problem that underwent a discretization process of dividing the world into a grid. The problem involves an initial state, *I*, and two potential goal states, *A* and *B*. As proven in (Kaminka et al., 2018), “given any cell size there exists a goal recognition problem such that goals may be indistinguishable in the discrete domain yet distinguishable in the continuous domain.” In the example, the trajectory of the agent clearly favors goal *A* (under the assumption of rationality) and could potentially lead to early recognition. However the discretized observation sequence of grid cells, does not convey this information. Figure 1, right, shows the same example illustrated as a state diagram of a 3-state FSM. The circles represent states and the arrows represent transitions between the states. In order for the agent to transition from the initial state, *I* to either state *A* or state *B* the agent would actually have to reach the state, making earlier recognition impossible. Therefore these representations of behaviours are not useful for capturing the progress between states.

A different approach to plan recognition, and one that has evolved to work well on continuous domains, involves utilizing a *domain theory* as part of the recognition process (Aineto et al., 2019; Pereira et al., 2019; Masters and Vered, 2021; Meneguzzi and Pereira, 2021). Plan recognition based on domain-theories assumes that any valid sequence of actions is a possible plan and using the domain description in the recognition process to generate possible plan hypotheses, thereby removing the reliance on a plan-library. Lesh and Etzioni (Lesh and Etzioni, 1995) and Martin et al. (Martin et al., 2015) compute specialized representations for goal recognition, constructed from a domain theory and incoming observations. Baker et al. use policies to calculate goal likelihoods by marginalizing over possible actions and generating state transitions, using only limited replanning (Baker et al., 2005). Pereira et al. use the domain theory to compute planning landmarks, and then use them to rank goal hypotheses and speed up the recognition process (Pereira and Meneguzzi, 2016; Pereira et al., 2017; Vered et al., 2018). However, all of these approaches work with discrete domain theories, and do not directly translate to continuous domains.

We are inspired by *Mirroring* (Vered et al., 2016; Vered and Kaminka, 2017), a goal recognition approach, especially suited towards continuous domains and most closely related to *plan recognition as planning* (PRP) which was first presented by Ramırez and Geffner (2010, 2009) and further developed by Sohrabi et al. (2016), and Masters and Sardina (2019). In PRP, the recognizer uses an unmodified planner as a black box to generate recognition hypotheses that match the observations. A heuristic comparison between the generated plans and an optimal plan that ignores the observations is used to probabilistically rank the hypotheses. Inspired by these investigations, *Mirroring* uses a planner (as a black box) to generate plan and goal hypotheses on-the-fly over continuous domains. While this approach is adequately suited towards complex, continuous domains, it has only been used in the context of *goal* recognition and needs to be uniquely adapted to be able to recognize behaviours.

Behaviour recognition in beyond visual range (BVR) air combat has also been much researched. Borck et al. (2015b) used case based behaviour recognition. The set of actions the agent attempts to recognize are: pursuit, whereby an agent flies directly at another agent, drag, whereby an agent tries to kinematically avoid a missile by flying away from it, and crank, whereby an agent flies at the maximum offset but tries to keep its target in radar. In this work, they also assume full knowledge of the capabilities of each observed aircraft rather than obtaining that information through observations. Additionally, the adaption to continuous domains happens by discretizing the position and heading of each agent into the features of their evaluated cases. As shown by Kaminka et al. (2018), discretization could lead to reduced recognition accuracy because for every possible grid cell-size there exists a goal recognition problem that is indistinguishable in the discrete domain, yet distinguishable in the continuous domain.

Borck et al. (2015a) also address the multi agent behaviour recognition problem in beyond visual range or BVR air combat by incorporating a planner to assist with the recognition process. A participating UAV performs simultaneous online behaviour recognition and planning to assist observation gathering tasks. However the main focus of this work was to study the relations between acting to influence the generated observation and the recognition inference. Their recognition process is very expensive as it requires a decomposition of tasks, *M* ^*k*^ candidate models for the planner, *M* being independent models for individual agents and *k* being the number of agents.

To address this we provide a formal definition to the problem of kinodynamic behaviour recognition which now enables us to perform multi agent behaviour recognition in BVR air combat scenarios through the use of an updated and adapted recognition algorithm, inspired by Mirroring, which leverages the power of the ACE0 simulator. In our work we use continuous planners to generate the possible behaviour hypotheses hence improving on the efficiency of the recognition process. Within the context of kinodynamic behaviour recognition this means that our approach can potentially leverage existing work on kinodynamic motion planning (Chen et al. (2020); Barbosa et al. (2021); Mohammed et al. (2021)) to generate potential plan hypotheses.

Many domains are brought together in this work, an analysis most similar to the goals of our work would be in the treatment of beyond visual range combat by (Floyd et al., 2017a,b). Floyd et al. (2017b) introduce the Tactical Battle Manager (TBM), which uses several integrated techniques to control an UAV in simulated air combat scenarios. The behaviour recognition component identifies the target aircraft by measuring the angle of each potential target vs the entity’s angle, again using a discretization process. The opponent with the lowest standard deviation over time is classified as the target, assuming the entity will keep its target within its field of vision. Each aircraft can either be *attacking* or *evading*. In contrast to their work, we focus primarily on combat within visual range using a range of planner-based behaviour recognition. We apply some of the techniques explored by Vered and Kaminka (2017) to a continuous flight behaviour recognition problem, successfully recognizing a range of behaviours simulated through the ACE0 multi-agent-based-simulation environment (Papasimeon and Benke, 2021) (described in further detail in Section 4).

## 3 Behaviour Recognition with Online Kinodynamic Planning

### 3.1 Online Kinodynamic Planning

We define the problem of *online kinodynamic planning* as the following optimization problem.

Definition 1 **(Online Kinodynamic Planning Problem).** *Given an initial state* $x_{start}\in X$ *, and a planning horizon* *T* *, the task is to find a policy* *π* = *u*_0_ *,* …, *u*_*t*_, …, *u*_*T*_ *, where* *u*_*t*_ ∈ *U*(*x*_*t*_) *for* *t* = 1, …, *T* − 1 *and disturbance* *w*_*t*_ ∈ *D*(*x*_*t*_, *u*_*t*_)*, that maximizes the cost*

$$\begin{array}{lll} \underset{u_{0},\ldots ,u_{T-1}}{\max} & E\left[\overset{T-1}{\underset{t=0}{\sum }}q\left(x_{t},u_{t},w_{t}\right)+p\left(x_{T}\right)\right] & \\ s.t.\; & x_{t+1}=f\left(x_{t},u_{t},w_{t}\right), & t=0,\ldots ,T-1\\ & x_{0}=x_{start} & \end{array}$$ (1)

$X\in R^{n}$ represents the set of possible states of the world in continuous spaces, in line with standard motion planning notation (LaValle, 2006). *U*(*x*_*t*_) are applicable actions or control inputs, *D*(*x*_*t*_, *u*_*t*_) is the random disturbance characterised by either an independent probability distribution or a distribution dependent only on the current state and control inputs, *q* is the *stage cost* and *p* is the terminal cost. Stage costs allow us to specify limits over the trajectory followed by the agent to ensure trajectories are smooth and velocities are not too high, ensuring trajectories impose less stress on the airframe. Terminal costs are used to favour trajectories that end up in states which are deemed to be useful for future trajectories. We note that we do not solve the problem in Definition 1 *optimally*, but rather we aim at finding policies $\hat{\pi }$ with costs that are less than or equal to that of π*, the *maximizer* or *optimal solution*, through the use of an off-the-shelf planner. flushleft

### 3.2 Kinodynamic Behaviour Recognition

We build on the plan recognition problem definition in (Kaminka et al., 2018) and update it to address behaviour recognition. We define the behaviour recognition problem *R* as:

$$R≔\left\langle X,O,x_{start},B\right\rangle$$ (2)

$X\in R^{n}$ represents the set of possible states of the world. *O* is defined as an *ordered sequence* of observations [*o*_1_, …, *o*_|*O*|_], where each observation *o*_*x*_ ∈ *O* is also a state $o_{x}\in X$ . $x_{start}\in X$ is the initial state and each behavior *β* = ⟨*q*, *p*⟩, *β* ∈ *B* is defined by the stage and terminal cost functions *q* and *p*. Each behavior is associated with a prior probability *P*(*β*). We refer to *actions* or control inputs as *u* ∈ *U*, and *plans* or policies as *π* ∈ *U* ^*T*^, where *T* is the horizon of the kinodynamic problem.

We define the *kinodynamic behavior recognition* problem as the task to determine β*, the behavior hypothesis with maximal probability, given the observation sequence.

Definition 2 **(Kinodynamic Behaviour Recognition).** *Given an observation sequence* *O* = [*o*_0_, …, *o*_*j*_, …, *o*_|*O*|_]*, where* *j* =  max(|*O*| − *T*, 0)*, and* *T* *is the horizon of the kinodynamic planning problem, the initial state* *x*_*start*_ = *o*_*j*_ *is set to the observed state* *T* *steps before the last observation, or the initial observation in the sequence if* *T* ≥|*O*|*. The online kinodynamic behavior recognition problem is defined as* $\beta^{*}={\mathrm{argmax}}_{\beta \in B}E_{\pi_{X}^{\beta }}\left[P\left(\pi_{X}^{\beta }\;|\;\left[o_{j},\ldots ,o_{\left|O\right|}\right]\right)\right]$ *, where* $\pi_{X}^{\beta }=\left[f\left(x_{t},u_{t},w_{t}\right)\;|\;t=0,\ldots ,T-1,\;u_{t}\in \pi^{\beta }\right]$ *is the sequence of states induced by the policy* *π* ^*β*^ *of the kinodynamic planning problem whose stage and cost functions are defined by* *β* *.*

Each policy *π* ^*β*^ is computed using Approximate Dynamic Programming (Bertsekas, 2017). The planner computes a T-step lookahead using Iterative Width (Lipovetzky and Geffner, 2012) to efficiently approximate the optimal policy (Ramirez et al., 2018). The available observations *O*_*T*_ = [*o*_max(|*O*|−*T*,0)_, …, *o*_|*O*|_] are matched against $\pi_{X}^{\beta }$ by taking their Euclidean distance or *L*_2_ norm:

$$d\left(O_{T},\pi_{X}^{B}\right)=\overset{\left|O_{T}\right|}{\underset{i=0}{\sum }}\|o_{i}-x_{i}{\|}_{2}$$ (3)

Where $x_{i}\in \pi_{X}^{\beta }$ and *o*_*i*_ ∈ *O*_*T*_. If |*O*_*T*_| < *T*, i.e., the sequence of states generated by $\pi_{X}^{\beta }$ is longer than the observation sequence, then the euclidean distance is computed only for the first |*O*_*T*_| observations. The likelihood of observing *O* given a behavior *β* is then defined with a Boltzmann distribution as follows:

$$P\left(O\;|\;\pi_{X}^{\beta }\right)=\frac{e^{-d\left(O,\pi_{X}^{\beta }\right)}}{\sum_{\beta^{\prime }\in B}e^{-d\left(O,\pi_{X}^{\beta^{\prime }}\right)}}$$ (4)

Behaviors that minimize the Euclidean distance with respect to the observations *O* are considered more likely, assuming agents are *perfectly rational* (Dennett, 2009) - as they approximate the optimal policy π*. The most likely behavior is then defined as P( $\pi_{X}^{\beta }\;|$ O) = *α* *P*(O $|\;\pi_{X}^{\beta }$ ) *P*(*β*), where *α* is a normalization factor, and P(*β*) is the prior probability of each behavior. In the experiments below, we assume a uniform prior distribution.

## 4 Simulating Aerial Maneuvers

### 4.1 Simulation Environment

To evaluate our behaviour recognition approach we utilize the state-of-the-art ACE multi-agent-based-simulation environment, which models multiple aircraft flying around a virtual environment. ACE includes computational representations of an aircraft’s flight dynamics, flight control systems and sensors. A number of agent-based models are used to represent the decision making models which control the aircraft, implementing higher level maneuvers and tactics. The agent-based decision making models can represent the reasoning processes of a human pilot or an autonomous system as part of an Unmanned Aerial Vehicle (UAV). For the purposes of this work, a light-weight version of ACE, known as ACE0 was employed. Further details on the architecture of ACE0 can be found in the paper by (Papasimeon and Benke, 2021). ACE0 is a minimal subset of ACE representing only two aircraft (or UAV) in 1v1 adversarial scenarios.

By default ACE0 implements both Finite State Machine (FSM) and Behaviour Tree (BT) based agent reasoning models to control the virtual aircraft in the simulation. More detail on behaviour trees can be found in the works of Martzinotto et al. (2014) and Colledanchise and Ögren (2017). However, ACE0 was specifically designed to accommodate different types of agent reasoning models. It has been used with a range of agent reasoning technologies, including automated planning (Ramirez et al., 2017, 2018), evolutionary algorithms (Masek et al., 2018; Lam et al., 2019; Masek et al., 2021), reinforcement learning (Kurniawan et al., 2019, 2020) and Generative Adversarial Networks (Hossam et al., 2020). In this work, we build upon the work of (Ramirez et al., 2017, 2018) using an automated hybrid planning approach combined with Model Predictive Control (MPC) to define and generate the behaviours in ACE0.

In ACE0 the full state *x*_*t*_ is defined by the state of each UAV *i*, represented by its position (*x*_*i*_, *y*_*i*_, *z*_*i*_), velocity vector (*v*_*xi*_, *v*_*yi*_, *v*_*zi*_) and orientation (*ψ*_*i*_, *θ*_*i*_, *ϕ*_*i*_), which correspond to the Euler angles representing the yaw, pitch and roll of the aircraft around the *z*_*i*_, *y*_*i*_ and *x*_*i*_ axes respectively. While ACE0 can support many different types of flight dynamics models of varying fidelity, in this study, a simplified flight dynamics model was used, with no random disturbance *w*_*t*_ and the following kinematic equations.

$$\dot{x}=v\left(t\right)\cos\psi \left(t\right)\cos\theta \left(t\right)\;,\;\dot{y}=v\left(t\right)\sin\psi \left(t\right)\cos\theta \left(t\right)\;,\;\dot{z}=v\left(t\right)\sin\theta \left(t\right)\;,\;\dot{\psi }=\frac{g}{v\left(t\right)}\tan\phi \left(t\right)$$ (5)

Here *v*(*t*) is the speed of the aircraft calculated as the magnitude of the velocity vector and *g* is the acceleration due to gravity. We assume the velocity vector is aligned with the aircraft’s nose and hence other aerodynamic effects such as the angle of attack aoa or *α*, the slide-slip angle *β* and the wind direction are not modelled.

Each UAV model in the simulation environment can be controlled through a number of *commanded* parameters (*ψ*_*c*_, *gload*_*c*_, *θ*_*c*_, *v*_*c*_). These represent the commanded yaw angle *ψ*_*c*_ which allows the aircraft to turn, the commanded g-load factor *gload*_*c*_ which affects the aircraft turn rate, the commanded pitch angle *θ*_*c*_ which allows the aircraft to climb and descend and the commanded speed *v*_*c*_ which allows the aircraft to speed up and slow down.

Hence, to turn to a desired heading *ψ*_*c*_ the pilot agent in the simulator can request a commanded yaw angle *ψ*_*c*_ together with a desired *gload*_*c*_ factor which will affect how fast the aircraft turns and as a result affects the roll angle *ϕ*. The pilot agent can also request the aircraft to climb or descend by requesting a commanded pitch angle *θ*_*c*_ or to speed up or slow down by requesting a desired speed *v*_*c*_. A simple flight control model within ACE0 interfaces with the flight dynamics model to control the UAV dynamics. The state of the system is hence defined by a state vector for each aircraft in the simulation. If we denote the blue and red aircraft as aircraft *i* and *j* we can present the state of the system *s*_*ij*_(*t*) at any given simulation time *t* as follows.

$$s_{ij}\left(t\right)=\left(\begin{array}{ccccccccc} x_{i}, & y_{i}, & z_{i}, & \psi_{i}, & \theta_{i}, & \phi_{i}, & v_{xi}, & v_{yi}, & v_{zi}\\ x_{j}, & y_{j}, & z_{j}, & \psi_{j}, & \theta_{j}, & \phi_{j}, & v_{xj}, & v_{yj}, & v_{zj} \end{array}\right)$$ (6)

Similarly, we can define the action vector *a*_*i*_(*t*), for aircraft *i* at simulation time *t* as follows;

$$\begin{array}{ccccc} a_{i}\left(t\right)= & \left(\psi_{ci},\right. & gload_{ci}, & \theta_{ci}, & \left.v_{ci}\right) \end{array}$$ (7)

Where the subscript *c* denotes a *commanded* or *desired* value. An overview of the UAV flight dynamics state variables and agent actions available in the simulator are shown in Table 1.

**TABLE 1:** An overview of the input state variables (left) and the commands available to control the simulated UAV in ACE0.

State Variables	Description	Agent Command	Parameters
*x*, *y*, *z*	Position	SetFlyLevelCmd	—
*ψ*, *θ*, *ϕ*	Orientation	SetPitchAngleCmd	*θ*_*c*_
*v*_*x*_, *v*_*y*_, *v*_*z*_	Velocity	SetAltitudeCmd	*z*_*c*_, *θ*_*c*_
*gload*	G-Load Factor	SetSpeedCmd	*v*_*c*_
*x*_*c*_, *y*_*c*_, *z*_*c*_	Commanded/Desired Position	SetHeadingCmd	*ψ*_*c*_
*ψ*_*c*_, *θ*_*c*_, *ϕ*_*c*_	Commanded/Desired Orientation	SetHeadingGLoadCmd	*ψ*_*c*_, *gload*_*c*_
*v*_*c*_	Commanded/Desired Speed	SetWaypointCmd	*x*_*c*_, *y*_*c*_
*gload*_*c*_	Commanded/Desired G-load		

These aircraft commands can be grouped together to construct higher level individual maneuvers, either stand alone or relative to another aircraft. These can include maneuvers to fly a particular search pattern and maneuvers relative to other aircraft such as intercepts and evasions. Subsequently these maneuvers can be used in Finite State Machines (FSM) or Behaviour Trees (BT) to implement more sophisticated tactical behaviour. However, as stated previously, in this work the behaviours are generated as sequences of actions in the form of plans generated by an automated planner.

**FIGURE 2:**
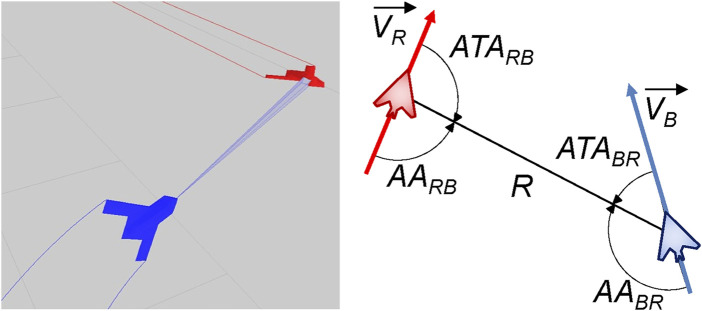
**(Left)** 3D visualisation of a two UAVs (blue and red) from a run generated by the ACE0 simulation environment. **(Right)** Relative angle conventions between the two UAV as implemented in ACE0.

When considering maneuvers relative to another aircraft a number of additional parameters must be considered, which can be seen in Figure 2. In this figure, the blue UAV has a sensor lock on the red UAV. The right image shows the relative orientation of the blue and red UAVs, which is defined through a number of attributes; From the perspective of the blue UAV, these are: *R*, the range between the two aircraft; *ATA*, the antenna train angle 1, the angle of the red aircraft relative to the blue aircraft’s nose; and *AA*, the aspect angle, the angle from the tail of the red aircraft relative to the blue aircraft. These angular conventions come from the domain of air combat defined by Shaw (1985) and Burgin and Sidor (1988) and can be used to define relative maneuvers and behaviours between aircraft.2 By definition (and as shown in Figure 2) these angles satisfy the following relationship.

$$AA_{BR}+ATA_{BR}=AA_{RB}+ATA_{RB}=\pi$$ (8)

From the perspective of the blue aircraft it is then possible to represent the simulation state at time using a set of derived state variables. We can describe this derived state as $s_{BR}^{\prime }\left(t\right)$ where

$$s_{BR}^{\prime }\left(t\right)=\left(ATA_{BR},AA_{BR},R_{BR},\Delta v_{BR}\right)$$ (9)

The distance or range between the blue and red aircraft is specified as *R*_*BR*_ and the velocity differential between blue and red is Δ*v*_*BR*_. This application of feature engineering to reduce to size of the state space to derived or domain relevant variables is common in machine learning applications. For example, this reduced feature set shown in Eq. 9 was used as the input to a reinforcement learning algorithm to discover agent policies for ACE0 in the work of Kurniawan et al. (2019, 2020).

**FIGURE 3:**
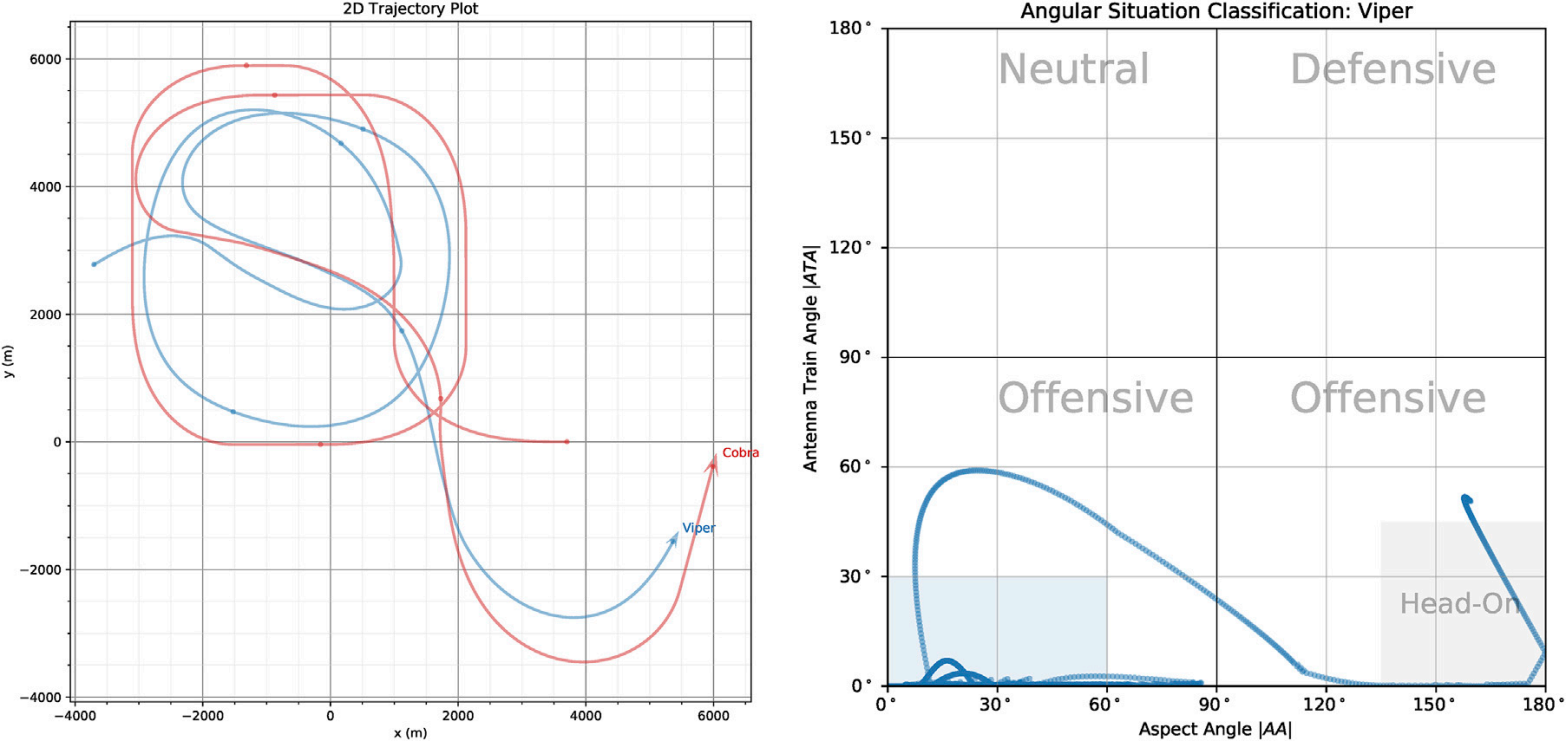
**(Left)** ACE0 generated trajectories of Viper (Blue UAV) and Cobra (Red UAV) in 2D-space. **(Right)** The angular situation of Viper relative to Cobra in orientation space where the *x*-axis plots the aspect angle |*AA*| against the antenna train angle |*ATA*| on the *y*-axis.

The *ATA* and *AA* angles are important, because in many military aircraft missions it is important to know how far off the nose an opponent aircraft is as well as if we are approaching it head-on (nose-to-nose) or approaching it from the rear sector. This is shown in Figure 3 which shows the trajectories two UAV, Viper (Blue) and Cobra (Red) in the 2D plane (left) and in orientation-space (right).

In this scenario, the red UAV (Cobra) flies through a series of way-points while the blue UAV (Viper) attempt to pursue Cobra. The angular situation diagram allows us to specify the tactical situation at any given time-step in the simulation run. According to common convention we plot the absolute values of the ATA and AA angles (Park et al., 2016). Also by convention, each of the quadrants is labelled according to the tactical posture the current aircraft (labelled in grey text in the figure), with the bottom two quadrants labelled as *Offensive* and the top two quadrants labelled as *Neutral* and *Defensive*. They are defined as follows:

• Offensive (Bottom-Right Quadrant) $\frac{\pi }{2}⩽|AA|⩽\pi$ and $0⩽|ATA|⩽\frac{\pi }{2}$ corresponding to the forward sectors of each aircraft being approximately aligned. If we further constrain the angular conditions to $\frac{\pi }{4}⩽|AA|⩽\pi$ and $0⩽|ATA|⩽\frac{\pi }{4}$ then we consider both aircraft to be *Head-On* which is indicated by the grey shaded area in the bottom-right quadrant of Figure 3.

• Offensive (Bottom-Left Quadrant) $0⩽|AA|⩽\frac{\pi }{2}$ and $0⩽|ATA|⩽\frac{\pi }{2}$ corresponding to the current aircraft pointing it’s nose to it’s opponent’s tail. For some maneuvers the goal is not only to get behind the opponent’s tail but to meet stricter angular parameters. This is indicated by the shaded blue region which is specified by $0⩽|AA|⩽\frac{\pi }{3}$ and $0⩽|AA|⩽\frac{\pi }{6}$ .

• Neutral (Top-Left Quadrant) $0\leq |AA|⩽\frac{\pi }{2}$ and $\frac{\pi }{2}⩽|ATA|⩽\pi$ corresponding to the two aircraft approximately facing away from each other.

• Defensive (Top-Right Quadrant) $\frac{\pi }{2}⩽|AA|⩽\pi$ and $\frac{\pi }{2}⩽|ATA|⩽\pi$ corresponding to the opponent aircraft aligning it’s nose with the current aircraft’s tail. In some situations (like air combat) this is considered dangerous and hence the aircraft is in a defensive posture.

For aircraft that are facing each other head-on, angular situation being in the bottom right hand corner of the angular situation plot, it is often the case that they want to maneuver themselves behind the opposing aircraft and find themselves in the bottom left hand corner of the angular situation plot. As mentioned above, the goal is satisfied once the aircraft is in blue shaded region shown in Figure 3 (Right).

This type of maneuver is important for a number of reasons, including visual identification of a threat or non-cooperative aircraft, formation flying and escort, aerial refuelling and in some instances, air combat where some weapons can only be employed against the rear sector, stern in naval terminology, of an opponent aircraft. As such, this type of maneuver is known as a *Stern Conversion*. In the following section we describe the stern conversion and other behaviours that our behaviour recognition system attempts to recognise.

### 4.2 Agent Behaviours

In this section we describe the five aerial maneuvers that we considered in our experimental study. These are:

• Stern Conversion. The observing agent executes a series of maneuvers to get behind the opponent aircraft while maintaining a specified separation distance on approach.
• Pure Pursuit. The observing agent points the aircraft nose to the opponent and flies directly at it.
• Lead Pursuit. The observing agent points the aircraft nose at a specified lead angle in front of the opponent and tries to fly a pursuit maneuver that leads the opponent. The idea here is to point the nose ahead of the opponent trajectory to predict where it is going.
• Offensive Flight. The observing agent attempts to keep the opponent in the two lower (offensive) quadrants of the angular situation plot.
• Fly Straight. The observing agent flies a straight line completely ignoring the opponent aircraft. We use this maneuver as a baseline.

All of these maneuvers are implemented in ACE0 in either low level pilot action or higher level behaviours which are composed of lower level actions.

Depending on the initial condition of the simulation (position, orientation and speed of the two aircraft) it is entirely possible that all these maneuvers (with the exception of the *Fly Straight* maneuver) can not only succeed in achieving the goal of maneuvering behind an opponent aircraft but could do so with some very similar looking trajectories. This being the case, the ability to distinguish between the behaviours is very challenging to achieve.

**FIGURE 4:**
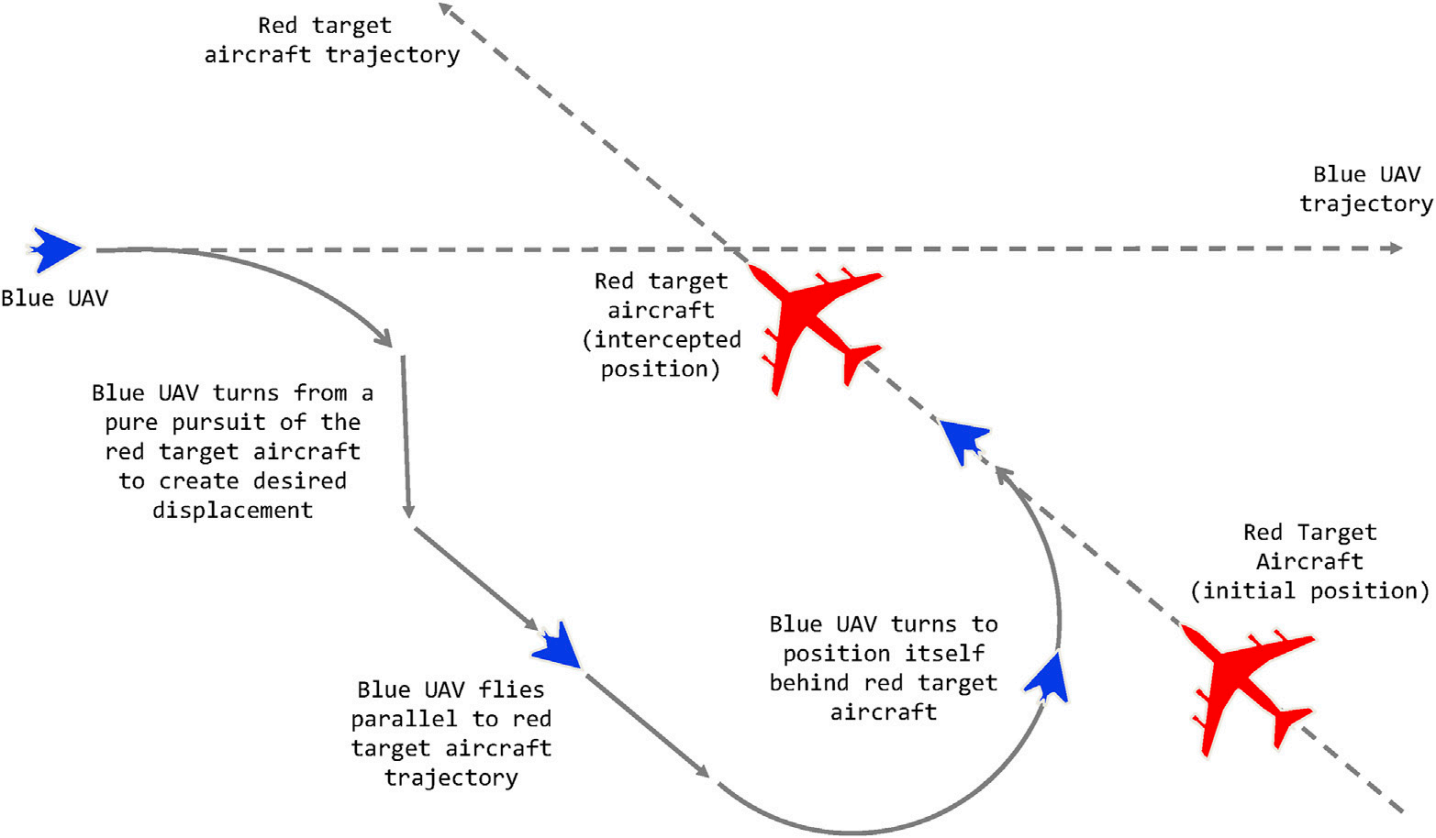
A conceptual diagram of a blue UAV (the observer) performing a stern conversion maneuver with respect to a larger red aircraft. Reproduced with permission from Papasimeon and Benke (2021).

A conceptual representation of a stern conversion can be seen in Figure 4. The maneuver is composed of a series of lower level actions and maneuvers. One can imagine if the blue aircraft started roughly behind the red aircraft the trajectory would look closer to a *Pure Pursuit* maneuver rather than the blue trajectory represented here.

**FIGURE 5:**
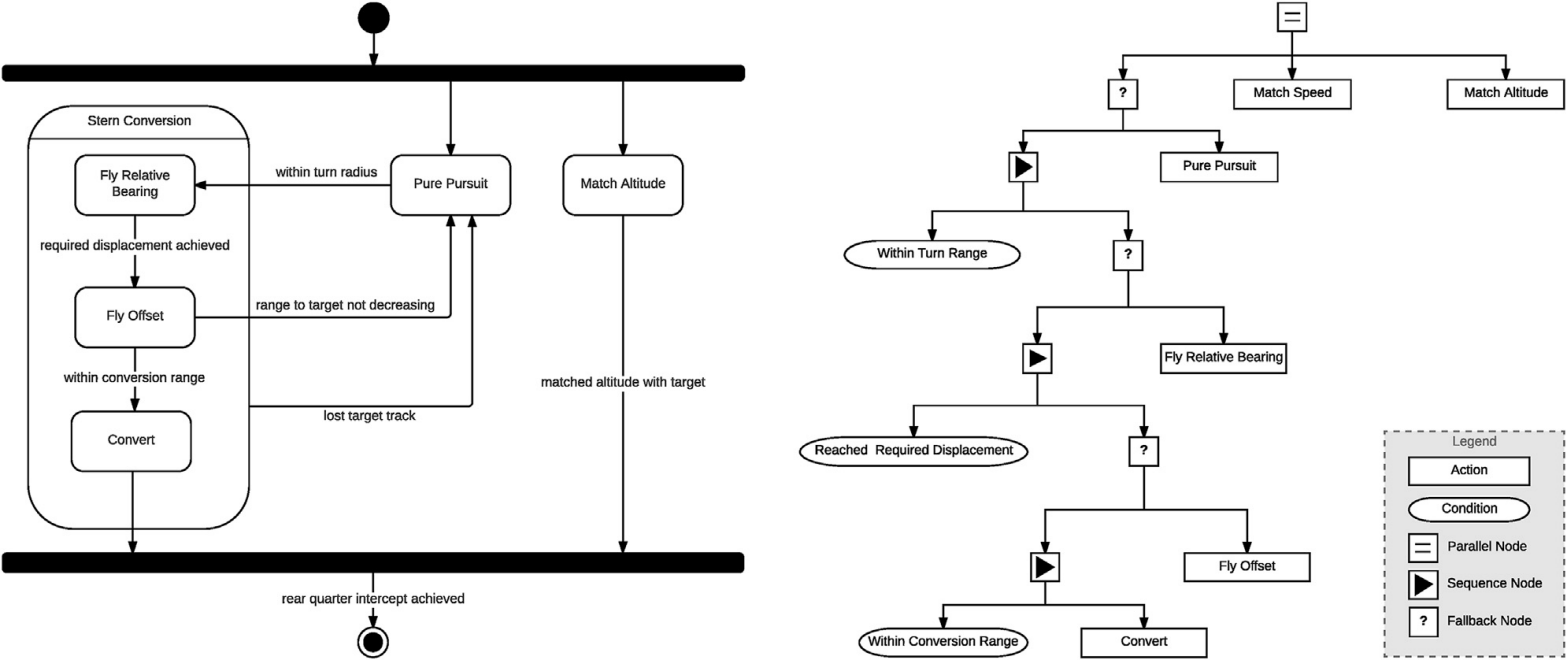
Agent diagrams from ACE0 implementing a stern conversion maneuver **(Left)** Stern conversion implement as a finite state machine (FSM) agent and **(Right)** Stern Conversion maneuver implemented as a behaviour tree (BT) agent. Reproduced with permission from Ramirez et al. (2017) and Ramirez et al. (2018).

Figure 5 presents graphical representations of the stern conversion maneuver. On the left is a finite state machine (FSM) representation of the decision logic, while on the right we have behaviour tree (BT) representation of the same maneuver. In the FSM representation the maneuver is made up of a series of states which execute lower level maneuvers and events allowing the FSM to transition from one state to another. As can be seen, this representation is rather simplistic such that at any point in time the agent can only be in one of five states. In the behaviour tree implementation the execution of behaviours is determined by the state of the conditional nodes in the tree. While more complex and flexible than the FSM representation, this representation still lacks the flexibility of a continuous plan generation. By using an automated planner to generate the stern conversion maneuver we allow for a more flexible representation that makes possible the recognition of complete behaviour trajectories according to highly sensitive cost functions.

**FIGURE 6:**
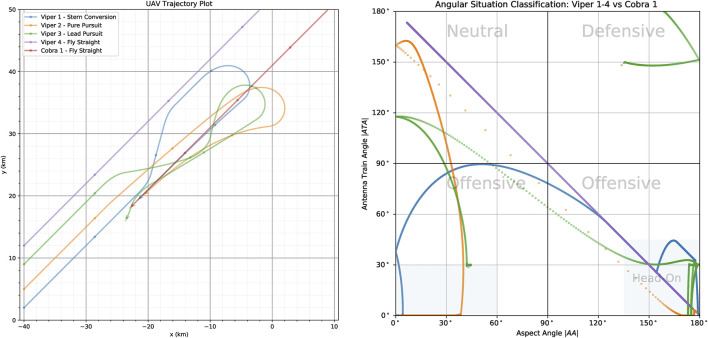
Comparison of behaviours generated by ACE0, showing four aircraft flying different behaviours with respect to the red aircraft (Cobra-1) which is implementing *Fly Straight*.

Figure 6 shows indicative trajectories for some of the behaviours being considered. There are five aircraft in this scenario; Viper-1 (Blue), Viper-2 (Orange), Viper-3 (Green), Viper-4 (Purple) and Cobra-1 (Red). The opponent aircraft all start from a slightly different starting location and implement the following behaviours; Viper-1 (Blue) is performing *Stern Conversion*; Viper-2 (Orange) performs *Pure Pursuit*; Viper-3 (Green) performs *Lead Pursuit*; Viper-4 is executing *Fly Straight* (Left) Trajectory of Cobra-1 and Vipers-1\enleadertwodots four in the 2D plane. In the right vignette in Figure 6 we can *see* corresponding trajectories in the angular situation classification space. The left chart in the figure shows the trajectories of all aircraft on the 2D plane, whereas the right chart shows the angular situation of Viper 1–4 each with respect to Cobra-1 (Red). The trajectories represent are generated from a 300 s (5 min) ACE0 simulation run. Cobra-1 (Red) starts the scenario in the top-right hand corner of the grid and flies in a straight line at a heading of 225°, with its trajectory crossing the map diagonally.

On the other hand, Viper-1,2,3,4 all start on the opposite side of the map with a heading of 45° effectively facing Cobra-1 and flying in its general direction. Viper-1,2,3,4 all start from the same x-coordinate but are staggered by a couple of kilometres in the initial y-coordinate for the purposes of clarity.

Viper-1 flies a *Stern Conversion* maneuver indicated by the Blue trajectory. As can be seen by the Blue trajectory, the stern conversion involves Viper-1 flying towards opponent aircraft, Cobra-1. Once a specified range is reached, the maneuver begins and Viper-1 flies at a specified offset angle until a desired horizontal separation has been achieved. Once they have passed each other, Viper-1 begins a turn to get behind Cobra-1 and begins to approach it to follow it. We can see that this has been successfully achieved both in the trajectory chart and in the angular situation classification chart, where Viper-1 starts in the bottom right corner (effectively head-on) and manages to maneuver the situation to the bottom-left hand corner where its behind Cobra-1’s tail. As one can imagine, the shape of the stern conversion maneuver is susceptible to the initial conditions of the two aircraft. For example, an attempt at a stern conversion will look more like pursuit if the two aircraft are not facing head-on initially.

In comparison if we look at the trajectory for Viper-2 (Orange) we simply have behaviour that “chases” Cobra-1. If we look at both charts from Figure 6 we can see at some point, due to the relative speeds of Viper-2 and Cobra-1, that Viper-2 passes Cobra-1 and ends up in the Neutral situation (with tails facing each other), hence requiring Viper-2 to turn around and chase Cobra-1; ultimately achieving the goal of getting behind it.

On the other hand Viper-3 (Green) is flying a *Lead Pursuit* maneuver, where it attempts to maintain a lead angle of Cobra-1 (Red). We can see from the angular situation chart that it manages to succeed with the transition from head-on to following Cobra-1. We can see that this maneuver causes a slight overshoot at times ending up in neutral territory, settling at a lead angle of |*ATA*| ≈ 30° for a short time it overtakes Cobra-1 and ends up in a defensive position for a short period of time at the end of the simulation run. This problem could be alleviated by making the maneuver slightly more complicated through the introduction of speed control. Currently both aircraft fly at their initial speed.

Viper-4 (Purple) flies in a straight line, slightly offset from a direct face-to-face encounter with Cobra-1. However, the story of Viper-4’s trajectory with respect to Cobra-1 is easily told by the situation classification chart where it starts in the lower right offensive quadrant proceeds to end up in the top-left neutral quadrant with the two aircraft tails pointing at each other.

As can be seen in Figure 6 the *Stern Conversion* maneuver maintains an angular situation classification of an offensive posture, that is within the lower two quadrants, for the entirety of the maneuver. As such, this maneuver can also be considered a valid *Offensive Flight* maneuver.

In the following we describe the behaviours as implemented from the perspective of the planning based behaviour recognition system, including formal definitions of the goal and cost functions.

### 4.3 Behaviour Specifications

#### 4.3.1 Notation and Definitions

We use **p**_*b*_ and **p**_*r*_ to denote the position vectors and **V**_*b*_ and **V**_*r*_ to denote the velocity vectors of the blue and red aircraft respectively. The distance (or range) between the two aircraft is then defined as

$$R=|p_{b}-p_{r}|$$ (10)

We use *R*_*min*_ and *R*_*max*_ to specify the minimum and maximum range. The antenna train angle (ATA) and the aspect angle (AA) described earlier are now defined as

$$ATA=\left[\arccos\left(\frac{\left(p_{r}-p_{b}\right)\cdot v_{b}}{\left\|p_{r}-p_{b}\|\;\|v_{b}\right\|}\right)\right]$$ (11)

And

$$AA=\left[\pi -\arccos\left(\frac{\left(p_{b}-p_{r}\right)\cdot v_{r}}{\left\|p_{b}-p_{r}\|\;\|v_{r}\right\|}\right)\right]$$ (12)

We use the notation *ATA*_*ub*_ and *AA*_*ub*_ to denote upper bounds on the *ATA* and *AA* angles respectively.

In the following we describe each of the behaviours being considered; *Stern Conversion* (sc), *Pure Pursuit* (pp), *Lead Pursuit* (lp), *Offensive Flight* (of) and *Fly Straight* (fs). Each of the behaviours is formally defined in terms of a scoring function *S*_x_ where the subscript x denotes the behaviour. The cost and and terminal functions *q* and *p* that were described in Section 3.2 are specified in terms of each behaviour’s scoring function.

$$q\left(x,u\right)=p\left(x\right)=S_{x},\forall x\in \left\{\text{SC},\text{PP},\text{LP},\text{OF},\text{FS}\right\}$$ (13)

For each behavior, functions *q* and *p* follow the same definition.

#### 4.3.2 Stern Conversion

The purpose of the *Stern Conversion* behaviour is for the blue UAV to maneuver itself behind the red UAV such that a number of conditions are met. These are that |*ATA*| ≤ *ATA*_*ub*_, |*AA*| ≤ *AA*_*ub*_ and *R*_*min*_ ≤ *R* ≤ *R*_*max*_. The exact values of these parameters depend on the purpose of attempting a stern conversion (such as obtaining a visual identification, air to air refuelling, formation flying or putting the blue aircraft into an offensive zone). In this work we use the values of *ATA*_*ub*_ = 60°, *AA*_*ub*_ = 30°, *R*_*min*_ = 100 m and *R*_*max*_ = 6000 m, which represent the conditions for a rear quarter visual identification of the red UAV. The scoring function for the stern conversion maneuver *S*_sc_ is defined as

$$S_{\text{sc}}=w_{g}\;g_{pa}+\left(1-w_{g}\right)\;\rho_{sc}$$ (14)

Where *w*_*g*_ is a constant weight balancing the reward of maintaining the goal condition and orienting towards the goal. We set this value to 0.8. *g*_*pa*_, the score given for satisfying the goal is defined as

$$g_{pa}=\left\{\begin{array}{cc} 1.0,\; & \text{if }\begin{array}{c} R_{min}\leq R\leq R_{max}\;and\\ |AA|<AA_{ub}\;and\\ |ATA|<ATA_{ub} \end{array}\\ 0.0,\; & \text{otherwise} \end{array}\right.$$ (15)

Where *AA*_*ub*_ is $\frac{\pi }{6}\left(30{}^{\circ}\right)$ , *ATA*_*ub*_ is $\frac{\pi }{3}\left(60{}^{\circ}\right)$ , *R*_*min*_ is 100 m, *R*_*max*_ is 6000 m. We specify the reward score as *ρ* so as to not overload the symbol *R* which is used to specify the range between the two aircraft. The reward score for the stern conversion behaviour *ρ*_*sc*_ is made up an angular orientation component and a range component. This means in order to maximise the score both the relative angular orientation and the range between the two aircraft need to be considered.

$$\rho_{sc}=\frac{1}{2}\left\{\frac{1}{2}\left[\left(1-\frac{\left|AA\right|}{\pi }\right)+\left(1-\frac{\left|ATA\right|}{\pi }\right)\right]+\exp\left[-\frac{2\left(R-R_{d}\right)}{\pi k_{R}}\right]\right\}$$ (16)

We used a *k*_*R*_ constant value of 1,000 m and a *R*_*d*_ value of 1,000 m. *R*_*d*_ denotes the desired range we want to be behind the opponent aircraft.

#### 4.3.3 Pure Pursuit

A *Pure Pursuit* behaviour involves pointing the aircraft’s nose at a specific target and flying directly towards it. It can be considered a *following* behaviour as it simply follows the designated target. This can be implemented by setting the desired heading of the UAV to the *ATA* of the target. This is a common behaviour that can be used for various reasons from pursuing an opponent, to formation flying, visual identification and refueling. The scoring function for the pure pursuit maneuver *S*_pp_ is

$$S_{\text{pp}}=k_{r}\;\exp\left[-\frac{k}{\pi }\left(\pi -\left|\right.|\psi_{b}-\xi |-\pi \left|\right.\right)\right]$$ (17)

Where *k*_*r*_ and *k* are set to a constant value of 10, *ψ*_*b*_ is the yaw of the controlled, blue, aircraft and *ξ* is the pure pursuit angle and is defined as

$$\xi =\arctan\left[\frac{y_{r}-y_{b}}{x_{r}-x_{b}}\right]$$ (18)

Where (*x*_*b*_, *y*_*b*_) and (*x*_*r*_, *b*_*r*_) are the (*x*, *y*) cartesian coordinates of the blue controlled and red aircraft respectively.

#### 4.3.4 Lead Pursuit

A *Lead Pursuit* behaviour points the blue aircraft’s nose a certain number of degrees in front of the red aircraft. A lead angle of *ψ* ^+^ = 30° is commonly used. This is known as *pulling lead* and attempts to predict where the red aircraft will be in the future and hence is used in intercept maneuvers. We implemented this behaviour by rewarding actions that get the UAV closer to a heading which points 30° in front of the opposing UAV. The scoring function for the lead pursuit maneuver *S*_lp_ is

$$S_{\text{LP}}=k_{r}\;\exp\left[-\frac{k}{\pi }\left(\pi -\left|\right.|\psi_{b}-\eta |-\pi \left|\right.\right)\right]$$ (19)

With the same constant values used in the *Pure Pursuit* behaviour. We denote the lead heading using *η* and define it as follows

$$\eta =\left\{\begin{array}{cc} \xi +\psi^{+},\; & \xi^{\prime }>\xi \\ \xi -\psi^{+},\; & \xi^{\prime }<\xi \\ \xi ,\; & \text{otherwise} \end{array}\right.$$ (20)

Where *ψ* ^+^ is the lead angle, which we set to a constant value of $\frac{\pi }{6}$ , and *ξ*′ is the look ahead lead angle defined as

$$\xi^{\prime }=\arctan\left[\frac{y_{r}^{\prime }-y_{b}^{\prime }}{x_{r}^{\prime }-x_{b}^{\prime }}\right]$$ (21)

Where $y_{r}^{\prime }$ , $y_{b}^{\prime }$ , $x_{r}^{\prime }$ and $x_{b}^{\prime }$ are

$$x_{b}^{\prime }=x_{b}+k^{\prime }v_{xb}y_{b}^{\prime }=y_{b}+k^{\prime }v_{yb}$$ (22)

$$x_{r}^{\prime }=x_{r}+k^{\prime }v_{xr}y_{r}^{\prime }=y_{r}+k^{\prime }v_{yr}$$ (23)

We use a *k*′ constant value of 5.0, standing for 5 s of lookahead. *v*_*xb*_ and *v*_*yb*_ are the (*x*, *y*) components of the controlled aircraft’s current velocity and *v*_*xr*_ and *v*_*yr*_ are the (*x*, *y*) components of the enemy aircraft’s current velocity.

#### 4.3.5 Offensive Flight

If a blue aircraft can maneuver to always keep the red aircraft *in front of it*, it typically means it has the advantage. This is known as being in an *offensive*, as opposed to a defensive, posture and is defined as keeping the *ATA* between ± 90°. We implemented this behaviour by rewarding actions which put the UAV in a state where the antenna train angle from the UAV to the opposing UAV is between ± 90°, awarding a higher score the closer the antenna train angle is to zero. The scoring function for the offensive flight maneuver *S*_OF_

$$S_{\text{OF}}=k_{r}\;\exp\left[-\frac{k}{\pi }\left(\pi -\left|\right.|\psi_{b}-\omega |-\pi \left|\right.\right)\right]$$ (24)

Where the offensive heading angle, *ω*, is defined as

ω=ATA (25)

Where as mentioned previously *ATA* is the antenna train angle and *ψ*_*b*_ is the heading of the controlled blue aircraft.

#### 4.3.6 Fly Straight

The *Fly Straight* behaviour aims for the UAV to fly straight and level. We included this behaviour for two reasons. First, it is the simplest behaviour and may therefore be used as a baseline and second, it is used as a building block for more complex behaviours. As a result it can be easily confused with other behaviours. We implemented this behaviour by providing a negative reward to actions that may change the direction of the UAV. The scoring function for the fly straight maneuver *S*_FS_ is

$$S_{\text{FS}}=-|g_{load}|$$ (26)

Where *g*_*load*_ is the g force load on the controlled aircraft while it is undertaking turning with a roll angle of *ϕ*.

## 5 Experimental Setup

### 5.1 Experimental Architecture

The experimental architecture used in this work involves a two stage process; a behaviour *generation* process which generates aircraft trajectories to be used as observations and a behaviour *recognition* process which attempts to recognise and correctly classify observations to behaviours.

**FIGURE 7:**
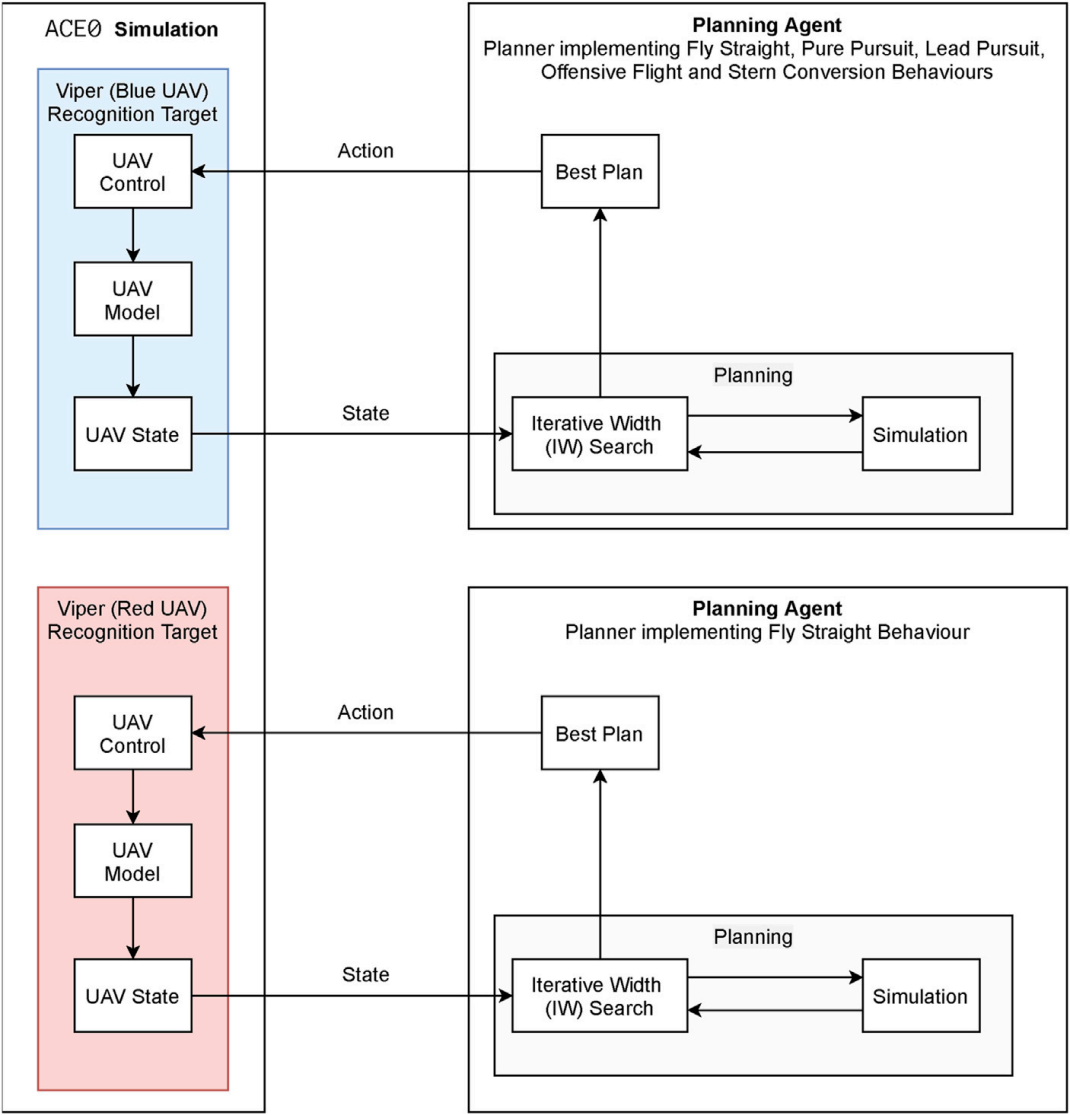
Behaviour generation framework.

The high level overview of the behaviour *generation* process is shown in Figure 7 which also illustrates the relationship between the ACE0 simulation environment and multiple autonomous planners. As can be seen the ACE0 behaviour simulation encompasses the behaviour of two aircraft (UAV), *Viper* (denoted in blue) and *Cobra* (denoted in red). The behaviours for both aircraft are controlled by automated planners. In this example *Cobra*, the red UAV, always flies in a straight line and *Viper*, the blue UAV, flies one of the five behaviours mentioned previously (Fly Straight, Pure Pursuit, Lead Pursuit, Offensive Flight and Stern Conversion). This is the process used in order to generate observations for recognition.

**FIGURE 8:**
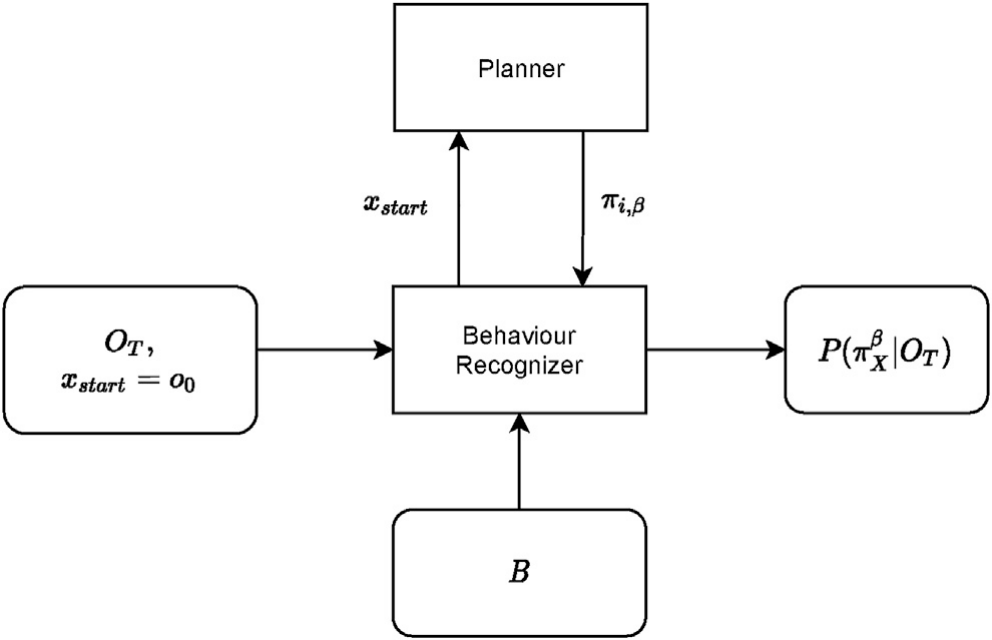
Behaviour recognition framework.

The high level overview of the behaviour *recognition* process is shown in Figure 8. This is the process whereby *Cobra*, the red UAV, aims to accurately recognize the behaviour of *Viper*, the blue UAV. The recognizer receives as input the last *T* steps of the observation sequence, and the set of possible behaviours *B*, defined in Section 3.2. The behaviour recognizer initial state *x*_*start*_ is set to the beginning of the observation sequence. The recognizer then utilizes a planner for generating potential behaviour sequences which it will compare against the observation sequence, at the end of which process the recognizer will output the posterior probability of the behaviours given the observation sequence, Section 3.2, Eq. 3.

### 5.2 Experimental Configurations

To evaluate our behaviour recognition approach we need to consider a range of different initial configurations for the blue and red UAVs. The primary factors that affect the initial configuration is the range *R* (distance) between the UAVs and their relative orientation as shown in Figure 2. By varying the relative headings of the two UAVs we obtain different values for the antenna train angle (*ATA*) and the aspect angle (*AA*). In all configurations we set the red UAV flying straight and level (that is in a straight line) at a fixed altitude and speed. To obtain our different experimental configuration we vary the blue UAV’s range and angular orientation relative to the red UAV. Hence from the perspective of the blue UAV each experimental configuration can be specified with three values (*R*, *ATA*, *AA*). We use a horizon of T = 10 timesteps with a timestep of *δ* = 0.1 s to define the kinodynamic problem (Def. 1). The planner solves the kinodynamic problem on every new state as it uses only the first applicable action *u*_0_ of the returned policy. The new state results from the application of the action through the simulator.

As a part of experimental exploration, smaller experiments were performed to investigate different values of T, the search horizon, ranging from T = 1 to T = 100. these different experiments produced minor different behaviours. These may represent more optimal local or more optimal global states considered by the planner, but as we only consider the control actions from the first state suggested by the planner, a large lookahead tends to be significantly less important in deriving real-time, online planning decisions. The timestep resolution, *δ*, similarly generates behaviours with minor differences, but smaller scale informal tests suggest this likely does not meaningfully change the recognition properties of the algorithm and probably isn’t a particularly promising area to examine.

**FIGURE 9:**
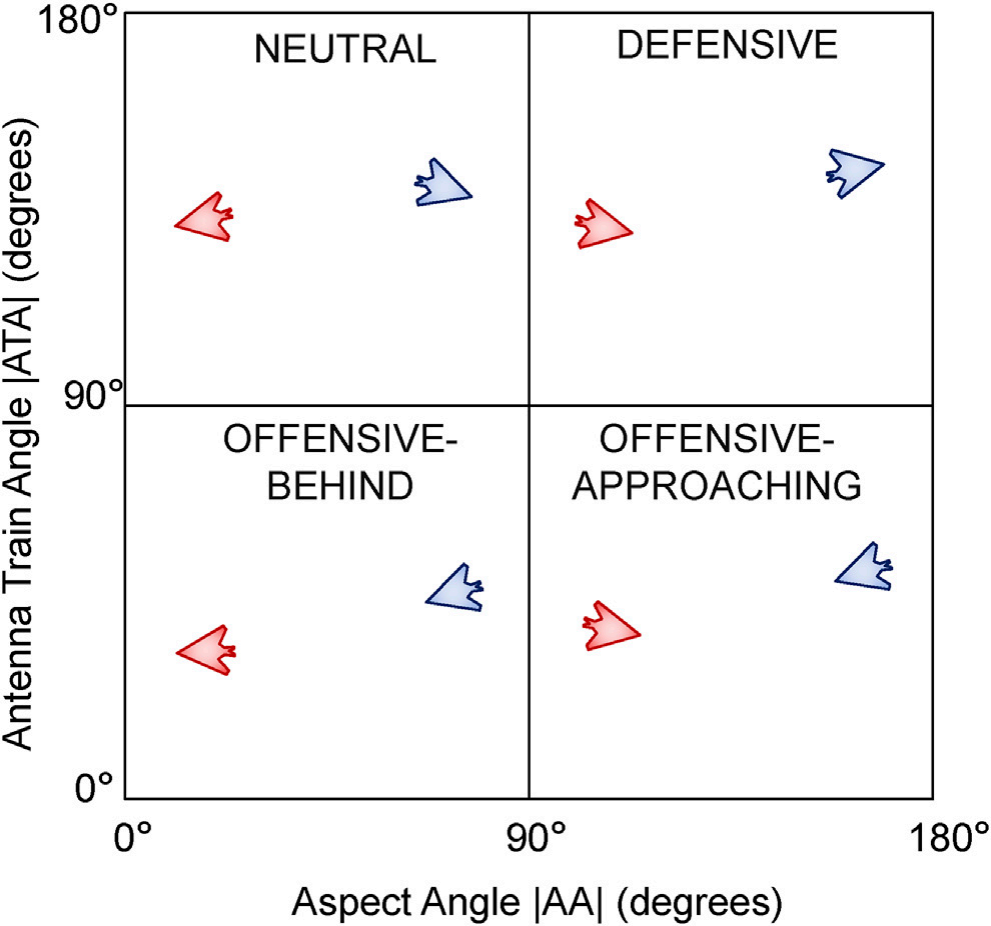
Relative orientation configurations defining the Blue UAV’s posture relative to the Red UAV.

We consider 36 starting starting positions for the blue UAV on a 6 × 6 grid at 2.5 km increments up to a maximum of ±10 km in the *x* and *y* axes relative to the red UAV. For each of these 36 starting positions for blue, we consider four representative relative orientations. We define these relative orientations using the conventions of the air combat domain (Burgin and Sidor, 1988; Park et al., 2016) where the absolute value of the aspect angle |*AA*| is plotted against the absolute value of the antenna train angle |*ATA*| as shown in Figure 9. The convention is to take advantage of symmetry, hence both axes are in the range of 0°–180°. Any point on this chart represents a unique relative orientation of the blue UAV relative to the red UAV. However we consider four representative cases as shown in Figure 9 by splitting the chart in to four quadrants each one 90 ° × 90 ° in size. Starting in the top left quadrant, we define these as follows:

• Neutral: The blue and red UAVs are generally facing away from each other and are in a neutral relative posture.
• Defensive: The blue UAV has its tail facing the red UAV and is hence in a defensive posture.
• Offensive-Approaching: Both blue and red UAVs have their noses roughly facing and approaching each other and the blue UAV is in an offensive position. Also known as a *head-on* configuration.
• Offensive-Behind: The blue UAV is behind the red UAV’s tail also in an offensive posture or configuration.

In many air combat scenarios, one of the goals of a pilot during a mission may be to maneuver their aircraft from the situation represented by the bottom right hand corner (facing head-on with an opponent) in Figure 9 into a configuration represented by the bottom left hand corner (getting behind an opponent).

Given our 36 starting locations and our four relative orientations we then have 144 geometric configurations in our experimental setup. Finally, given these initial starting conditions we consider the five aerial maneuvers that we want the blue UAV to fly and are the subject of our behaviour recognition experiment. We consider the *Fly Straight*, *Pure Pursuit*, *Lead Pursuit*, *Offensive Flight* and the *Stern Conversion* maneuvers. This gives us a total of 36 × 4 × 5 = 720 scenarios to consider. With the exception of the straight line maneuver, all maneuvers flown by the blue UAV are flown relative to the red UAV.

We consider a range of positions and orientations because the maneuvers the blue UAV flies will look different depending on the initial configuration. In fact, in many cases we expect that some maneuvers will look very similar to others making them difficult to distinguish not only by automated behaviour recognition algorithms but also by human experts.

### 5.3 Evaluation

We used the following metrics to evaluate the performance and efficiency of our behaviour recognition algorithm:

• True Positive Rate The number of *time steps* the correct hypothesis was ranked as most likely (i.e., rank 1), which indicates general accuracy. The more frequently the recognizer ranked the correct hypothesis first, the more reliable we consider the recognizer to be. We again normalize using the length of the observation sequence.

• Single True Positive Rate The number of time steps the correct hypothesis was ranked as most likely *alone*. By this measure we are able to completely identify the chosen behaviour, with no ties or confusion with other behaviours. This indicates a higher level of confidence and precision by the recognizer. The more frequently the recognizer solely ranked the correct hypothesis first, the more confident it is. Higher values indicate better recognition success. We again normalize using the length of the observation sequence.

• False Positive Rate The number of *time steps* a behaviour which was not the true behaviour was ranked first. Since our approach allows for multiple behaviours to be ranked first concurrently, Section 3.2 Eq. 3, this metric is of critical importance in determining the similarity of trajectories and the quality of discrimination. For example, a trivial behaviour recognition technique would be to assume all behaviours are the true behaviour, without considering the false positive rate; this will rank well in other metrics, despite not being useful in practice, if not outright dangerous. We further evaluated this measure by looking at the *number of behaviours* tied as leading behaviour hypotheses as well as *which of the behaviours* were tied.

## 6 Results

### 6.1 Successful Behaviour Recognition

Our approach showed great promise for recognizing a range of complex aerial maneuvering behaviours. We achieved a *True Positive* rate of 100% over all behaviours. This means that we always included the correct behaviour in the set of highest ranked behaviour hypotheses. Since the observations tested were not noisy, we were able to accurately match the partial observations to behaviour hypotheses generated by the planners on demand. Complementary to this result we also achieved a *False Negative* rate of 0%, meaning we never excluded the correct behaviour from our goal hypotheses.

Distinguishing completely between behaviours, as can be determined by the *Single True Positive* rate, proved more challenging due to the complexity of the domain and the similarities between the behaviours. Even though, we were able to fully distinguish the correct behaviour in 434, i.e. 60% out of the initial 720 scenarios tested. For those scenarios which were distinguishable, the percent of observations whereby the correct behaviour was recognized *alone* in each scenario, can be viewed in Table 2.

**TABLE 2:** Behaviour recognition mean, minimum, maximum and standard deviation of true positive rates.

	Fly	Pure	Lead	Offensive	Stern
	Straight	Pursuit	Pursuit	Flight	Conversion
Mean	0.999	0.874	0.970	0.865	0.963
Min	0.980	0.495	0.660	0.496	0.495
Max	1.000	0.991	1.000	0.991	1.000
SD	0.004	0.126	0.053	0.136	0.099

As can be seen, the *Fly Straight* behaviour was the most recognizable behaviour, being uniquely distinguished over 99.9% of the observations on average per scenario, with a minimum value of 98% and a maximum value of 100%. This means that for each of the 434 scenarios where *Fly Straight* was the correct behaviour, we were able to recognize it as the only possible behaviour for almost all of the observation sequence. This was somewhat to be expected as the *Fly Straight* behaviour is independent of the trajectory of the opponent (red) aircraft with the direction being the only distinguishing feature.

The hardest behaviour to recognize proved to be the *Offensive Flight* behaviour. On average we were able to uniquely distinguish it from all other behaviours in 86.5% of the observation sequences, with a minimum value of 49.5% and a maximum value of 99.1%. The trajectory generated by the *Offensive Flight* behaviour is not only dependant on the trajectory of the opponent aircraft but is also highly dependent on the initial configuration. This behaviour attempts to maneuver the blue aircraft to position the red aircraft in its forward sector, hence it is not obvious (even for a human observer) what the trajectory should look like. For certain initial orientations (such as *Offensive-Behind*) the trajectory could resemble a *Pure Pursuit* or *Lead Pursuit* maneuver.

### 6.2 Potential Sources for Behaviour Confusion

**FIGURE 10:**
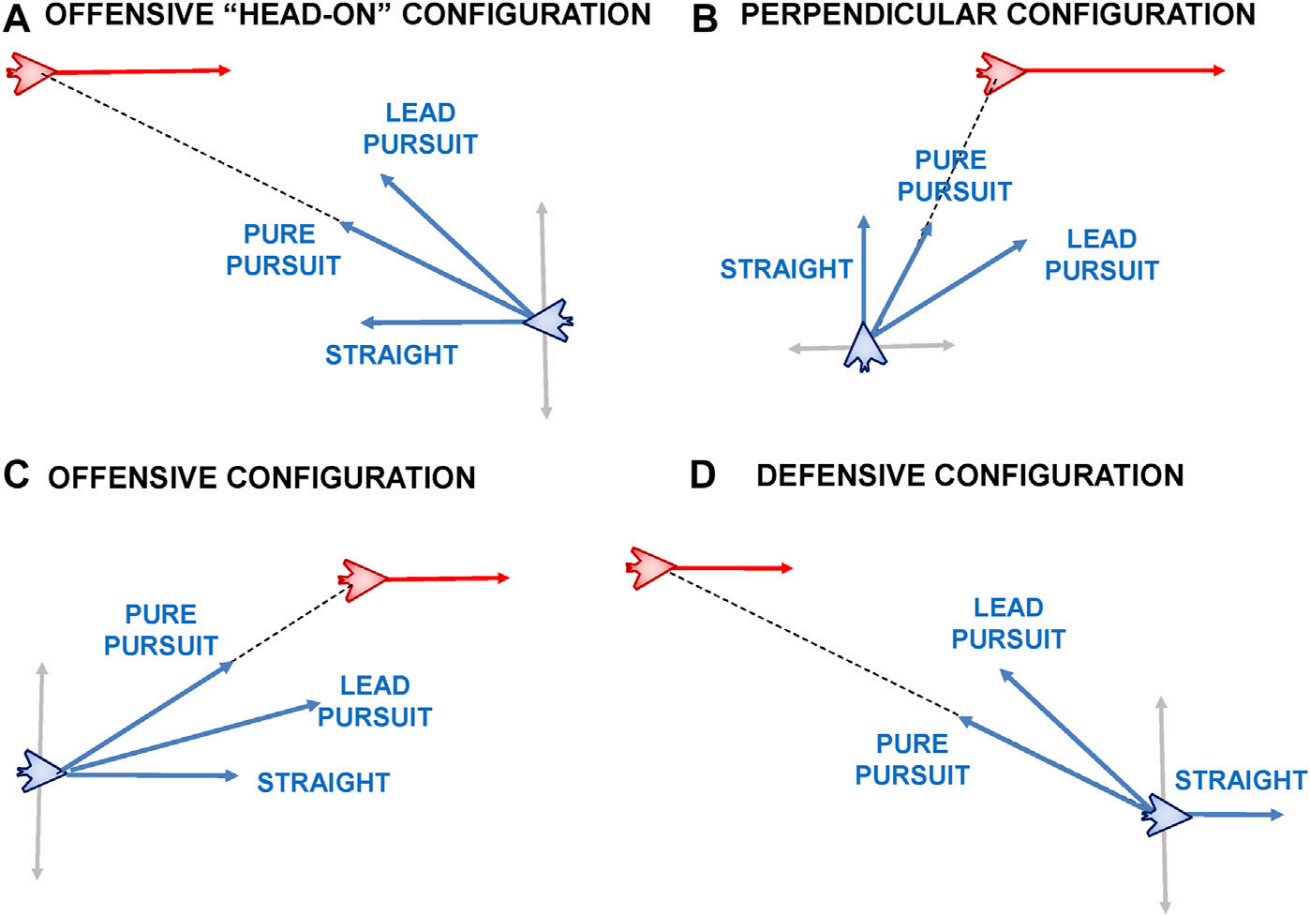
Behaviour similarities under different initial conditions which could lead to potential confusion in successful behaviour recognition.

One of the difficulties in distinguishing between the behaviours considered in this paper is that they often result in similar trajectories depending on the initial aircraft configuration. We can demonstrate this through some examples as shown in Figure 10 looking at different initial configurations between blue and red aircraft. For the purposes of simplicity we assume all red aircraft fly straight and level.

In Figure 10A we see that in the initial configuration both aircraft are roughly facing head-on. The blue vectors in the Figure denote the Fly Straight, Pure Pursuit and Lead Pursuit maneuvers. While there is potentially some difficulty in distinguishing between these maneuvers in this configuration, the situation becomes even more difficult as they also approximate the trajectory of an Offensive Flight and Stern Conversion maneuver. In an Offensive Flight maneuver the blue aircraft is required to keep the red aircraft in its forward sector (denoted by |*ATA*| ≤ *π*). This is shown by the grey line intersecting the blue aircraft. As can be seen, the Fly Straight, Pure Pursuit and Lead Pursuit all satisfy the conditions for the offensive maneuver in this configuration. Similarly when considering the stern conversion, these are all good starting points until sufficient displacement and range is achieved to turn the blue aircraft around and point it at the red aircraft’s tail. We see a similar situation in Figures 10B,C whereby a Fly Straight maneuver or a Pure Pursuit can be easily considered as a valid Offensive Flight or Stern Conversion maneuver simply because the red aircraft is in a Defensive position.

Finally, in the Defensive configuration shown in Figure 10D, it would be easier and more intuitive for a human to distinguish between a Fly Straight maneuver and the two Pursuit maneuvers. In the case of the Pursuit maneuvers the blue aircraft needs to conduct a significant left hand turn. However, this is similar behaviour that would be expected for an Offensive Flight or a Stern Conversion maneuver.

### 6.3 Tied Behaviour Recognition

In light of these potential challenges, we evaluated the ties between the different behaviours while independently looking at each of the four different initial configurations; 36 starting positions, five behaviours for each position for a total of 180 behaviour recognition scenarios per initial configuration. Even though we were able to fully distinguish the correct behaviour in 60% (434/720) of the configurations considered, it is important to analyse the cases where multiple behaviours were tied as the most likely behaviour hypothesis. In this section we look at the recognition success and tie rate for each of the four orientation configurations shown originally in Figure 9 and attempt to explain why we might have ties between multiple behaviours.

• Offensive-Approaching: No ties in 136/180 cases (76% recognition success rate).
• Defensive: No ties in 132/180 cases (73% recognition success rate).
• Offensive-Behind: No ties in 100/180 cases (56% recognition success rate).
• Neutral: No ties in 66/180 cases (37% recognition success rate).

The initial configurations which result in the highest recognition successes are the *Offensive-Approaching* and the *Defensive* configurations. This is because for all maneuvers (except for straight line), in most cases the blue aircraft has to conduct a significant turn to get behind the red aircraft when starting in these configurations. These maneuvers contain large turning circles which should be also intuitively recognisable for human observers.

When starting in the *Offensive-Behind* configuration, many of the success criteria for successfully executing a maneuver have already been satisfied (with the red aircraft being in the blue aircraft’s forward sector). Hence many of the maneuvers devolve into a pursuit, making it more difficult to distinguish between the two pursuit maneuvers (*Pure/Lead Pursuit*) and one of the more complex maneuvers such as *Offensive Flight* or *Stern Conversion*. As expected we are unlikely to be able to easily distinguish between the maneuvers because they don’t have to vary their trajectory much to meet their goal criteria.

In the final case, (*Neutral*), where the aircraft are facing away from each other, the blue aircraft needs to execute a 180° turn to change its posture from *Neutral* to *Offensive-Behind*. In all maneuver cases (except for *Fly Straight*) these are going to look very similar.

### 6.4 Analysis of Tied Behaviours

**FIGURE 11:**
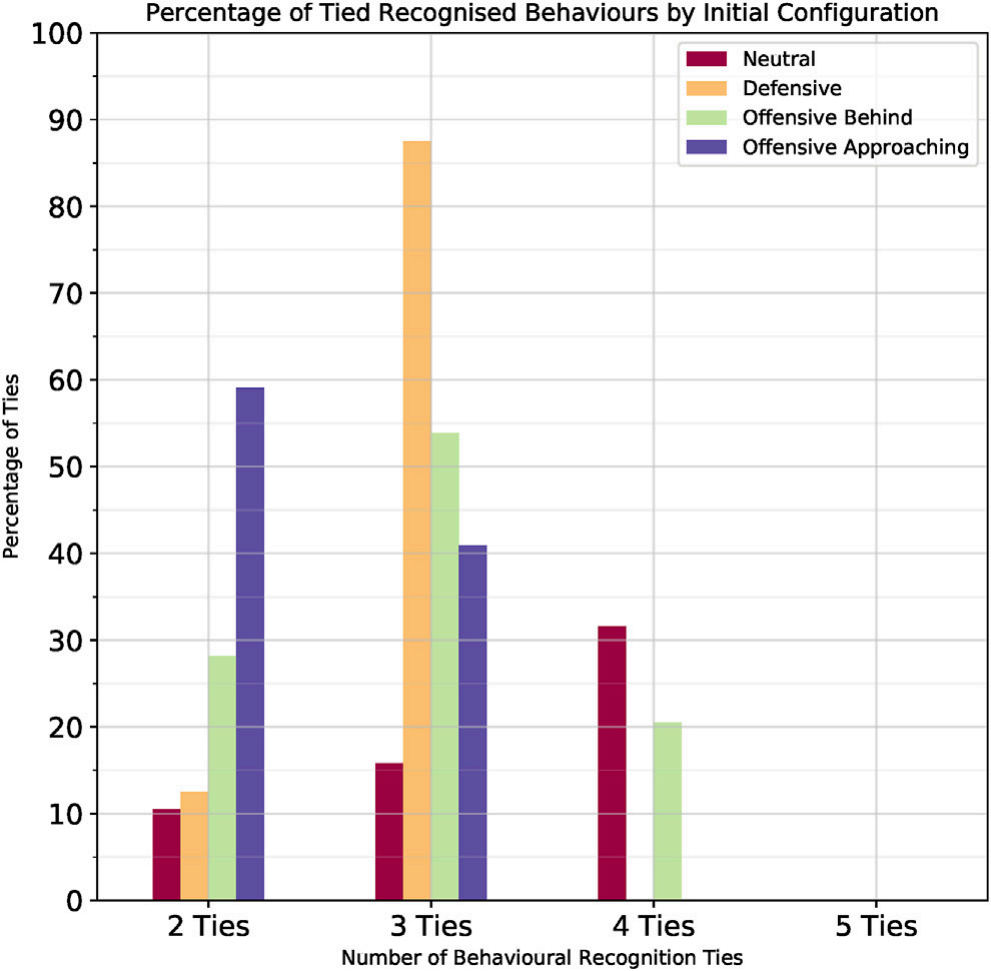
Percent of ties between behaviours per initial configuration.

We further analysed the behaviour hypotheses for those instances in which ties did occur. Figure 11 presents the percentage of how many behaviours were tied with regards to the different initial configurations. The *x*-axis represents how many behaviours were tied; the *y*-axis represents the percent out of all ties; and the different coloured bars represent the different initial configurations. Lower values are better, indicating less ties, hence a more confident recognition. As can be seen there were no 5-behaviour ties in any of the initial configurations. Hence, the recognizer was always able to rule out at least one behaviour. When looking at Table 3 it is apparent that the behaviour that was never tied with any other was the *Fly Straight* behaviour.

**TABLE 3:** The number of ties for each behaviour with respect to the different initial configurations, corresponding to the four quadrants of the angular situation classification.

	**Neutral**					**Defensive**				
	Fly Straight	Pure Pursuit	Lead Pursuit	Offensive Flight	Stern Conv	Fly Straight	Pure Pursuit	Lead Pursuit	Offensive Flight	Stern Conv
Fly	—	0	0	0	0	—	0	0	0	0
Straight										
Pure	0	—	66	114	84	0	—	42	47	0
Pursuit										
Lead	0	66	—	66	48	0	43	—	42	0
Pursuit										
Offensive	0	114	66	—	84	0	48	42	—	0
Flight										
Stern	0	84	48	84	—	0	0	0	0	—
Conv										

	**Offensive-behind**					**Offensive-approaching**				
	Fly Straight	Pure Pursuit	Lead Pursuit	Offensive Flight	Stern Conv	Fly Straight	Pure Pursuit	Lead Pursuit	Offensive Flight	Stern Conv
Fly Straight	—	0	0	0	0	—	0	0	0	0
Pure	0	—	16	78	57	0	—	0	44	18
Pursuit										
Lead	0	16	—	16	16	0	0	—	0	0
Pursuit										
Offensive	0	78	16	—	60	0	44	0	—	18
Flight										
Stern	0	58	16	60	—	0	18	0	18	—
Conv										

As explained in the previous section, the initial configuration in which there was most confusion was the *Neutral* configuration, with 114/180 instances, i.e. 73% of cases without conclusive recognition. Figure 11 shows a breakdown of those cases in terms of how many behaviours were tied. We can see that 31.58% of the ties occurred among four behaviours, 15.79% among three behaviours and 10.53% between two behaviours. This result is in agreement with our hypothesis that in all maneuver cases, except *Fly Straight*, these behaviours will look very similar.

In the *Offensive Behind* initial configuration, whereby 80/180 or 44% of the cases were not conclusively distinguishable, the majority of cases, 52.5%, arose because of ties among three behaviours. In the *Defensive* initial configuration, whereby most cases were conclusively distinguishable, over those 48 instances (27%) which weren’t, 87.5% of the ties were among three behaviours. Finally, in the *Offensive Approaching* initial configuration, over those 44 instances (24%) which were not conclusively distinguishable, 59.1% of the ties occurred between two behaviours. We looked into the specific tied instances and identified which of the behaviours were more easily confused with each other.

Table 3 presents the number of times each of the behaviours were tied to each other with respect to the different initial configurations. These ties were not unique and could have occurred along with other ties in accordance with Figure 11. The *Fly Straight* behaviour was easy to recognize and was not tied with any other behaviours. As previously stated this is because the blue aircraft flies in a straight line independent of the trajectory of the red aircraft. Intuitively, we also expect the *Pure Pursuit* and *Lead Pursuit* behaviours to be confused with each other because they are essentially the same behaviour with the only difference being that in the former the blue aircraft follows the red aircraft directly, whereas in the latter case the blue aircraft follows the red aircraft with a 30° angular lead. We can see this to be the case in three out of the four configurations in Table 3 where these two maneuvers are easily confused.

The only case where this doesn’t happen is in the *Offensive-Approaching* configuration where there is confusion between the *Offensive Flight* behaviour and the *Pure Pursuit* behaviour. This stands to reason, because in order for the goal conditions for *Offensive Flight* behaviour to be satisfied it needs to fly a trajectory that closely resembles a *Pure Pursuit*. We see this confusion between these two behaviours across all the initial configurations.

Finally we also expect some confusion in identifying a *Stern Conversion* maneuver for the *Offensive Flight* and *Pure Pursuit* behaviours. This is because when the two aircraft are facing each other all of these three maneuvers require a large turn to get behind the opponent aircraft, hence they look similar. In other initial configurations where a turn is not required, then a *Stern Conversion* devolves into a chase, making it look like a *Pure Pursuit*.

In the case where the two aircraft are facing head-on in the *Offensive-Approaching* configuration we can see confusion between *Stern Conversion* and *Offensive Flight* because in both cases the UAV has to fly a large turn trajectory to put it behind the opponent.

One of the challenges in our benchmarks was the similarity among different types of maneuvers which would make it difficult even for human experts to distinguish. Despite this, our approach performed well and we expect it to perform even better with more diverse behaviour sets.

## 7 Conclusion and Future Work

In summary we have formally defined the problem of *behaviour* recognition and developed a MPC, kinodynamic behaviour recognizer. We evaluated the performance of our recognizer in the challenging domain of aerial maneuvers and defined a set of benchmark aerial maneuver behaviours under a range of different initial configurations. We have chosen challenging, real-life maneuvers which may be partially similar in different parts of the trajectory, depending on the behaviour of the opposing aircraft as well as the initial geometrical configuration. We were able to achieve a True Positive rate of 100% and a False Negative rate of 0% while also evaluating the Single True Positive rates whereby the correct behaviour was recognized *alone*. This enabled us to provide more insight as to the similarities of the behaviours and the challenges of the aerial maneuver domain.

The ties between the recognition of some behaviours was expected due to a number of factors. First, was the similarity in the generated behaviour trajectories. For example, the difference between a *Pure Pursuit* and a *Lead Pursuit* behaviour is subtle, with only the lead angle being the only distinguishing feature. Second, more complex behaviours are composed of simpler behaviours. The *Stern Conversion* behaviour relies on the *Pure Pursuit* behaviour. This behavioural composability means that depending on the situation, a more complex behaviour begins to look like a simpler behaviour making them more difficult to distinguish and hence resulting in ties. Finally, the trajectories of the behaviours are not absolute. Rather they are highly context dependant with the resulting aircraft trajectories being influenced by the behaviour of the opposing aircraft and the initial geometric configuration. Being able to distinguish these types of aircraft maneuvers is difficult even for experienced pilots and air traffic controllers. This highlights either that the planner’s selected present behaviours which are fundamentally hard to discriminate or that the discrimination method is not sensitive to the differences. This is a very challenging domain that requires further study from the intention recognition research community.

In future we would like to incorporate additional features to the recognition process such as opponent intent, to further help distinguish between the different maneuvers and attempt to recognize sophisticated multi-aircraft maneuvers (such as formation flying). This can be achieved through the inclusion of a situation awareness model as was previously done for human decision making in complex tactical military scenarios (Vered et al., 2020).Also, and related, for any practical domain, it is be quite important to properly establish what the a priori probabilities of the behaviours are, for instance, depending on the tactical situation in the case of the air combat domain. We hypothesize that establishing these probabilities will significantly improve the performance of our approach.

Additionally, while we acknowledge that false positive rates are unavoidable and an inherent characteristic of any benchmark of scientific interest. More work needs to be done to provide assistance to human decision-makers to handle these when they happen. Perhaps recognition needs to be made aware of what the possible observer actions are, and to consider their potential outcomes to present a more nuanced classification of the sequence of observations.

Another possible angle to consider would be to extend the formulation of goal, plan and behaviour recognition to consider sets of goals, plans and behaviours rather than single goals, plans and behaviours. This hierarchical organisation of hypotheses would follow from, for instance, not considering classification errors between goals that belong to the same category as all that important. This is directly linked to the inclusion of multi-aircraft maneuvers, which opens up the possibility of exploring team behaviour recognition, an area of significant importance in many domains but expressively in the aerial maneuver domain. We would also like to compare our results against expert human recognition to facilitate human-agent teaming.

## Data Availability

The raw data supporting the conclusions of this article will be made available by the authors, without undue reservation.

## References

[B1] Aineto D. Jiménez S. Onaindia E. Ramírez M. (2019). Model Recognition as Planning. Proc. Int. Conf. Automated Plann. Scheduling 29, 13–21.

[B2] Azarewicz J. Fala G. Fink R. Heithecker C. (1986). “Plan Recognition for Airborne Tactical Decision Making,” in AAAI'86: Proceedings of the Fifth AAAI National Conference on Artificial Intelligence, 805–811. Available at: https://www.aaai.org/Papers/AAAI/1986/AAAI86-134.pdf .

[B3] Baker C. Saxe R. Tenenbaum J. B. (2005). “Bayesian Models of Human Action Understanding,” in Advances in Neural Information Processing Systems, Vancouver, BC, Canada, December 5–8, 2005, 99–106.

[B4] Barbosa F. S. Karlsson J. Tajvar P. Tumova J. (2021). “Formal Methods for Robot Motion Planning with Time and Space Constraints (Extended Abstract),” in International Conference on Formal Modeling and Analysis of Timed Systems (Springer), 1–14. 10.1007/978-3-030-85037-1_1

[B5] Bertsekas D. P. (2017). Dynamic Programming And Optimal Control. 4th Edn. Belmont, MA: Athena Scientific.

[B6] Borck H. Karneeb J. Aha D. W. Navy N. (2015a). “Active Behavior Recognition in beyond Visual Range Air Combat,” in Proceedings of the Third Annual Conference on Advances in Cognitive Systems ACS, 9.

[B7] Borck H. Karneeb J. Alford R. Aha D. W. (2015b). “Case-based Behavior Recognition in beyond Visual Range Air Combat,” in Twenty-Eighth International Flairs Conference.

[B8] Burgin G. H. Sidor L. B. (1988). Rule-Based Air Combat Simulation. Tech. Rep. 4160. National Aeronautics and Space Administration (NASA).

[B9] Calvanese D. De Giacomo G. Vardi M. Y. (2002). Reasoning about Actions and Planning in Ltl Action Theories. KR 2, 593–602. 10.1016/b978-0-08-051447-5.50007-4

[B10] Chen L. Mantegh I. He T. Xie W. (2020). “Fuzzy Kinodynamic Rrt: A Dynamic Path Planning and Obstacle Avoidance Method,” in 2020 International Conference on Unmanned Aircraft Systems (ICUAS) (IEEE), 188–195. 10.1109/icuas48674.2020.9213964

[B11] Colledanchise M. Almeida D. Ögren P. (2019). “Towards Blended Reactive Planning and Acting Using Behavior Trees,” in 2019 International Conference on Robotics and Automation (ICRA) (IEEE), 8839–8845. 10.1109/icra.2019.8794128

[B12] Colledanchise M. Ögren P. (2017). How Behavior Trees Modularize Hybrid Control Systems and Generalize Sequential Behavior Compositions, the Subsumption Architecture, and Decision Trees. IEEE Trans. Robot. 33, 372–389. 10.1109/TRO.2016.2633567

[B13] Dennett D. (2009). Intentional Systems Theory. The Oxford handbook Philos. mind, 339–350. 10.1093/oxfordhb/9780199262618.003.0020

[B14] Floyd M. W. Karneeb J. Moore P. Aha D. W. (2017a). “A Goal Reasoning Agent for Controlling Uavs in Beyond-Visual-Range Air Combat,” in Proceedings of the Twenty-Sixth International Joint Conference on Artificial Intelligence (IJCAI-17), 4714–4721. 10.24963/ijcai.2017/657

[B15] Floyd M. W. Karneeb J. Moore P. Aha D. W. (2017b). A Goal Reasoning Agent for Controlling Uavs in Beyond-Visual-Range Air Combat. IJCAI, 4714–4721. 10.24963/ijcai.2017/657

[B16] Sukthankar G. Goldman R. P. Geib C. Pynadath D. V. Bui H. (Editors) (2014). Plan, Activity, and Intent Recognition. San Francisco, CA: Morgan Kaufmann.

[B17] Heinze C. Cross M. Goss S. Josefsson T. Lloyd I. Murray G. (2002). Agents of Change: The Impact of Intelligent Agent Technology on the Analysis of Air Operations. chap. 6., 229–268. 10.1142/9789812776341_0006

[B18] Heinze C. Goss S. Pearce A. (1999). “Plan Recognition in Military Simulation: Incorporating Machine Learning with Intelligent Agents,” in Proceedings of IJCAI-99 Workshop on Team Behaviour and Plan Recognition, 53–64.

[B19] Heinze C. (2004). “Modelling Intention Recognition for Intelligent Agent Systems,” Ph.D. thesis (Melbourne, Australia: Department of Computer Science and Software Engineering, The University of Melbourne).

[B20] Heinze C. Papasimeon M. Goss S. Cross M. Connell R. (2008). “Simulating Fighter Pilots,” in Defence Industry Applications of Autonomous Agents and Multi-Agent Systems. Editors Pěchouček M. Thompson S. G. Voos H. (Basel: Birkhäuser Basel), 113–130.

[B21] Hossam M. Le T. Huynh V. Papasimeon M. Phung D. Q. (2020). “Optigan: Generative Adversarial Networks for Goal Optimized Sequence Generation,” in International Joint Conference on Neural Networks (IJCNN), Glasgow, UK, July 19–24, 2020. 10.1109/ijcnn48605.2020.9206842

[B22] Ilachinski A. (2004). Artificial War: Multiagent-Based Simulation of Combat. World Scientific.

[B23] Jiménez S. Segovia-Aguas J. Jonsson A. (2019). A Review of Generalized Planning. Knowledge Eng. Rev. 34. 10.1017/s0269888918000231

[B24] Kabanza F. Bellefeuille P. Bisson F. Benaskeur A. R. Irandoust H. (2010). “Opponent Behaviour Recognition for Real-Time Strategy Games,” in Plan, Activity, and Intent Recognition 10, 29–36.

[B25] Kaminka G. A. Vered M. Agmon N. (2018). Plan Recognition in Continuous Domains. AAAI, 6202–6210.

[B26] Kurniawan B. Vamplew P. Papasimeon M. Dazeley R. Foale C. (2019). “An Empirical Study of Reward Structures for Actor-Critic Reinforcement Learning in Air Combat Manoeuvring Simulation,” in AI 2019: Advances in Artificial Intelligence (Springer International Publishing), 54–65. 10.1007/978-3-030-35288-2_5

[B27] Kurniawan B. Vamplew P. Papasimeon M. Dazeley R. Foale C. (2020). “Discrete-to-deep Supervised Policy Learning: An Effective Training Method for Neural Reinforcement Learning,” in ALA 2020: Adaptive Learning Agents Workshop at AAMAS 2020 (Auckland, New Zealand.

[B28] Lam C.-P. Masek M. Kelly L. Papasimeon M. Benke L. (2019). A Simheuristic Approach for Evolving Agent Behaviour in the Exploration for Novel Combat Tactics. Operations Res. Perspect. 6, 100123. 10.1016/j.orp.2019.100123

[B29] LaValle S. M. (2006). Planning Algorithms. Cambridge, UK: Cambridge University Press.

[B30] Lesh N. Etzioni O. (1995). . IJCAI 95, 1704–1710. 10.5555/1643031.1643119

[B31] Lipovetzky N. Geffner H. (2012). “Width and Serialization of Classical Planning Problems,” in European Conference on Artificial Intelligence (ECAI).

[B32] Martin Y. E. Moreno M. D. R. Smith D. E. (2015). “A Fast Goal Recognition Technique Based on Interaction Estimates,” in Proceedings of the International Joint Conference on Artificial Intelligence (IJCAI), 761–768.

[B33] Marzinotto A. Colledanchise M. Smith C. Ögren P. (2014). “Towards a Unified Behavior Trees Framework for Robot Control,” in proceedings of 2014 IEEE International Conference on Robotics and Automation (ICRA 2014), 5420–5427. 10.1109/icra.2014.6907656

[B34] Masek M. Lam C. P. Benke L. Kelly L. Papasimeon M. (2018). “Discovering Emergent Agent Behaviour with Evolutionary Finite State Machines,” in PRIMA 2018: Principles and Practice of Multi-Agent Systems. Editors Miller T. Oren N. Sakurai Y. Noda I. Savarimuthu B. T. R. Cao Son T. (Tokyo, Japan: Springer International Publishing)), 19–34. 10.1007/978-3-030-03098-8_2

[B35] Masek M. Lam C. P. Kelly L. Benke L. Papasimeon M. (2021). “A Genetic Programming Framework for Novel Behaviour Discovery in Air Combat Scenarios,” in Data and Decision Sciences in Action 2. Editors Ernst A. T. Dunstall S. García-Flores R. Grobler M. Marlow D. (Cham: Springer International Publishing)), 263–277. 10.1007/978-3-030-60135-5_19

[B36] Masters P. Sardina S. (2019). Cost-based Goal Recognition in Navigational Domains. jair 64, 197–242. 10.1613/jair.1.11343

[B37] Masters P. Vered M. (2021). “What’s the Context? Implicit and Explicit Assumptions in Model-Based Goal Recognition,” in Proceedings of the Thirtieth International Joint Conference on Artificial Intelligence (IJCAI-21).

[B38] Ménager D. Choi D. Floyd M. W. Task C. Aha D. W. (2017). Dynamic Goal Recognition Using Windowed Action Sequences. Workshops at the Thirty-First AAAI Conference on Artificial Intelligence.

[B39] Meneguzzi F. Pereira R. F. (2021). “A Survey on Goal Recognition as Planning,” in Proceedings of the Thirtieth International Joint Conference on Artificial Intelligence (IJCAI-21). 10.24963/ijcai.2021/616

[B40] Mohammed H. Romdhane L. Jaradat M. A. (2021). RRT*N: an Efficient Approach to Path Planning in 3D for Static and Dynamic Environments. Adv. Robotics 35, 168–180. 10.1080/01691864.2020.1850349

[B41] Mohmed G. Lotfi A. Pourabdollah A. (2020). Enhanced Fuzzy Finite State Machine for Human Activity Modelling and Recognition. J. Ambient Intell. Hum. Comput 11, 6077–6091. 10.1007/s12652-020-01917-z

[B42] Oh H. Kim S. Shin H.-S. Tsourdos A. White B. A. (2014). Behaviour Recognition of Ground Vehicle Using Airborne Monitoring of Unmanned Aerial Vehicles. Int. J. Syst. Sci. 45, 2499–2514. 10.1080/00207721.2013.772677

[B43] Papasimeon M. Benke L. (2021). “Multi-Agent Simulation for AI Behaviour Discovery in Operations Research,” in 22nd International Workshop on Multi-Agent-Based Simulation (MABS 2021) (London, UK).

[B44] Park H. Lee B.-Y. Tahk M.-J. Yoo D.-W. (2016). Differential Game Based Air Combat Maneuver Generation Using Scoring Function Matrix. Int. J. Aeronaut. Space Sci. 17, 204–213. 10.5139/ijass.2016.17.2.204

[B45] Pereira R. F. Fuggitti F. De Giacomo G. (2021). Recognizing Ltlf/pltlf Goals in Fully Observable Non-deterministic Domain Models. arXiv [Preprint]. Available at: https://arxiv.org/abs/2103.11692 (Accessed March 22, 2021). 10.1007/s10489-023-05087-1 PMC10787694 38225993

[B46] Pereira R. F. Meneguzzi F. (2016). “Landmark-Based Plan Recognition,” in Proceedings of the European Conference on Artificial Intelligence (ECAI).

[B47] Pereira R. F. Oren N. Meneguzzi F. (2017). “Landmark-Based Heuristics for Goal Recognition,” in Proceedings of the Association for the Advancement of Artificial Intelligence (AAAI).

[B48] Pereira R. F. Vered M. Meneguzzi F. R. Ramírez M. (2019). “Online Probabilistic Goal Recognition over Nominal Models,” in Proceedings of the 28th International Joint Conference on Artificial Intelligence 10.24963/ijcai.2019/770

[B49] Ramırez M. Geffner H. (2009). “Plan Recognition as Planning,” in International Joint Conference on Artifical Intelligence, 1778–1783.

[B50] Ramırez M. Geffner H. (2010). “Probabilistic Plan Recognition Using Off-The-Shelf Classical Planners,” in International Joint Conference on Artificial Intelligence.

[B51] Ramirez M. Papasimeon M. Benke L. Lipovetzky N. Miller T. Pearce A. R. (2018). “Integrated Hybrid Planning and Programmed Control for Real Time Uav Maneuvering,” in 17th International Conference on Autonomous Agents and Multiagent Systems (AAMAS 2018) (Stockholm, Sweden), 1318–1326.

[B52] Ramirez M. Papasimeon M. Benke L. Lipovetzky N. Miller T. Pearce A. R. (2017). “Real–time Uav Maneuvering via Automated Planning in Simulations,” in 26th International Joint Conference on Artificial Intelligence (IJCAI) (Melbourne, Australia), 5243–5245.

[B53] Rao A. Artificial A. Murray G. (1994). “Multi-agent Mental-State Recognition and its Application to Air-Combat Modelling,” in 13th International Workshop on Distributed Artificial Intelligence.

[B54] Rao A. S. Georgeff M. P. (1991). “Modeling Rational Agents within a Bdi-Architecture,” in Proceedings of the Second International Conference on Principles of Knowledge Representation and Reasoning (San Francisco, CA: Morgan Kaufmann Publishers Inc.), 473–484.

[B55] Schmidt C. F. Sridharan N. S. Goodson J. L. (1978). The Plan Recognition Problem: An Intersection of Psychology and Artificial Intelligence. Artif. Intelligence 11, 45–83. 10.1016/0004-3702(78)90012-7

[B56] Shaw R. L. (1985). Fighter Combat. Annapolis, MD: Naval Institute Press.

[B57] Sitanskiy S. Sebastia L. Onaindia E. (2020). Behaviour Recognition of Planning Agents Using Behaviour Trees. Proced. Computer Sci. 176, 878–887. 10.1016/j.procs.2020.09.083

[B58] Sohrabi S. Riabov A. V. Udrea O. (2016). “Plan Recognition as Planning Revisited,” in The Twenty-Fifth International Joint Conference on Artificial Intelligence, 3258–3264.

[B59] Srivastava S. Immerman N. Zilberstein S. (2011). A New Representation and Associated Algorithms for Generalized Planning. Artif. Intelligence 175, 615–647. 10.1016/j.artint.2010.10.006

[B60] Van-Horenbeke F. A. Peer A. (2021). Activity, Plan, and Goal Recognition: A Review. Front. Robotics AI 8, 106. 10.3389/frobt.2021.643010 PMC8141730 34041274

[B64] Vered M. Howe P. Miller T. Sonenberg L. Velloso E. (2020). Demand-Driven Transparency for Monitoring Intelligent Agents. IEEE Trans. Hum. Mach. Syst. 50, 264–274.

[B61] Vered M. Kaminka G. A. Biham S. (2016). “Online Goal Recognition through Mirroring: Humans and Agents,” in The Fourth Annual Conference on Advances in Cognitive Systems.

[B62] Vered M. Kaminka G. A. (2017). “Heuristic Online Goal Recognition in Continuous Domains,” in Proceedings of the Twenty-Sixth International Joint Conference on Artificial Intelligence (IJCAI-17), 4447–4454. 10.24963/ijcai.2017/621

[B63] Vered M. Pereira R. F. Kaminka G. Meneguzzi F. R. (2018). “Towards Online Goal Recognition Combining Goal Mirroring and Landmarks,” in Proceedings of the 19th International Conference on Autonomous Agents and Multiagent Systems, Suécia, 2018.

